# TALEN-Based *HvMPK3* Knock-Out Attenuates Proteome and Root Hair Phenotypic Responses to flg22 in Barley

**DOI:** 10.3389/fpls.2021.666229

**Published:** 2021-04-29

**Authors:** Tomáš Takáč, Pavel Křenek, George Komis, Pavol Vadovič, Miroslav Ovečka, Ludmila Ohnoutková, Tibor Pechan, Petr Kašpárek, Tereza Tichá, Jasim Basheer, Mark Arick, Jozef Šamaj

**Affiliations:** ^1^Department of Cell Biology, Centre of the Region Haná for Biotechnological and Agricultural Research, Faculty of Science, Palacký University Olomouc, Olomouc, Czechia; ^2^Laboratory of Growth Regulators, Palacký University and Institute of Experimental Botany, Czech Academy of Sciences, Olomouc, Czechia; ^3^Institute for Genomics, Biocomputing and Biotechnology, Mississippi Agricultural and Forestry Experiment Station, Mississippi State University, Starkville, MS, United States; ^4^Laboratory of Transgenic Models of Diseases, Institute of Molecular Genetics of the CAS, Vestec, Czechia

**Keywords:** flagellin, barley, *HvMPK3*, TALEN, proteomics, root hairs, PR proteins, chitinases

## Abstract

Mitogen activated protein kinases (MAPKs) integrate elicitor perception with both early and late responses associated with plant defense and innate immunity. Much of the existing knowledge on the role of plant MAPKs in defense mechanisms against microbes stems from extensive research in the model plant *Arabidopsis thaliana*. In the present study, we investigated the involvement of barley (*Hordeum vulgare*) MPK3 in response to flagellin peptide flg22, a well-known bacterial elicitor. Using differential proteomic analysis we show that TALEN-induced *MPK3* knock-out lines of barley (*HvMPK3* KO) exhibit constitutive downregulation of defense related proteins such as PR proteins belonging to thaumatin family and chitinases. Further analyses showed that the same protein families were less prone to flg22 elicitation in *HvMPK3* KO plants compared to wild types. These results were supported and validated by chitinase activity analyses and immunoblotting for HSP70. In addition, differential proteomes correlated with root hair phenotypes and suggested tolerance of *HvMPK3* KO lines to flg22. In conclusion, our study points to the specific role of HvMPK3 in molecular and root hair phenotypic responses of barley to flg22.

## Introduction

Pathogens trigger innate immune mechanisms in plants by virtue of two different pathways. One depends on the perception of microbe-derived molecular patterns (called also pathogen-associated molecular patterns, PAMPs) and it is called PAMP-triggered immunity (PTI; reviewed in Peng et al., 2018). The second is related to the intracellular functions of microbe-delivered effectors and it is called effector-triggered immunity (ETI; reviewed in Cui et al., 2015). In the first case, PTI responses are a typical part of receptor-mediated signaling (e.g., Lee et al., 2019), while ETI responses may arise from the targeting of host proteins with structural and signaling roles by bacterial effector proteins (Lee et al., 2012; Cheong et al., 2014). Studies of PTI have been very much simplified by the identification of molecular patterns within microbial elicitors, such as flg22 and the prokaryotic elongation factor EF-Tu (Alhoraibi et al., 2019). Flg22 is perceived by a selective pattern recognition receptor, which is the leucine-rich repeat receptor kinase (LRR-RK) called FLAGELLIN SENSING 2 (FLS2; Gómez-Gómez and Boller, 2000; Robatzek and Wirthmueller, 2013). It has been shown that a minimal ligand for FLS2 is a 22 amino acid long peptide flg22, which is conserved in different flagellins (Felix et al., 1999; Gómez-Gómez et al., 1999). The events that follow perception of flg22 by FLS2 include its heterodimerization with another LRR-RK named BAK1 (BRI1-associated kinase 1; Chinchilla et al., 2007), the internalization of the receptor complex through ligand- (Robatzek et al., 2006), clathrin- (Mbengue et al., 2016) or sterol-dependent endocytosis (Cui et al., 2018), and the activation of downstream proteins such as BIK1 (Botrytis-induced kinase 1; Lu et al., 2010). Although members of flg22 perception and signal transduction are conserved among dicots and monocots (Robatzek et al., 2007; Takai et al., 2008; Mueller et al., 2012; Wang et al., 2015; Hao et al., 2016), large scale transcriptomic examination showed considerable rate of divergence between *Arabidopsis thaliana* and rice in regard to defense-related genes (Movahedi et al., 2011).

The defense response triggered by flg22 encompasses several measurable early onset events, including an oxidative burst with the accumulation of H_2_O_2_ via NADPH oxidases (e.g., Kadota et al., 2015), the activation of mitogen-activated protein kinase (MAPK) species (Rasmussen et al., 2012), the reorganization of the actin cytoskeleton and occasionally the disruption of cortical microtubules (reviewed in Henty-Ridilla et al., 2013; Li and Staiger, 2018). Such events may be coupled, or exerted independently of each other as was proven for oxidative stress and MAPK activation (Xu et al., 2014). PTI and ETI culminate to the transcriptional reprogramming resulting in expression of *PATHOGENESIS RELATED* (*PR*) genes (Frei dit Frey et al., 2014; Li et al., 2016). Importantly, activation of MAPKs holds a key transcriptional transactivation role since it regulates the activity of transcription factors responsible for the expression of PR genes (Meng and Zhang, 2013). In this line, several WRKY transcription factors have been identified to be targeted by MAPKs in different plant species, such as *Arabidopsis* (Sheikh et al., 2016) and *Nicotiana benthamiana* (Adachi et al., 2015). From numerous studies in *Arabidopsis*, four different MAPK species have been implicated in PTI, namely MPK3, MPK6, MPK4, and MPK11 (Asai et al., 2002; Suarez-Rodriguez et al., 2007; Bethke et al., 2011). MPK4 may be activated through a module encompassing the MAPK kinase kinase called MEKK1 (Suarez-Rodriguez et al., 2007) and MAPK kinases named MKK1 and MKK2 (Mészáros et al., 2006; Gao et al., 2008; Qiu et al., 2008). MPK3 and MPK6 are activated in parallel to MPK4 after flg22 perception and require MKK4/MKK5 pair for their activation (Asai et al., 2002). Although the responsible MAPK kinase kinase (MAPKKK) is not yet identified, some studies suggest it might be the ANP group (ARABIDOPSIS HOMOLOGUE OF NUCLEUS AND PHRAGMOPLAST ASSOCIATED KINASE; Asai et al., 2002; Savatin et al., 2014; Gigli Bisceglia et al., 2017), or MAPKKK3/MAPKKK5 (Sun et al., 2018). At least MPK3 has a positive role in PTI since the expression of its constitutively active variant (CA-MPK3; Genot et al., 2017) upregulates the transcription of *PR* genes in the absence of pathogen, or PAMP stimulation. In some instances, MPK3 and MPK6 function in a pairwise manner (e.g., Su et al., 2013) to regulate aspects of PTI such as the phosphorylation of transcription factors like BRASSINOSTEROID INSENSITIVE1-ETHYL METHANESULFONATE-SUPPRESSOR1 (BES1; Kang et al., 2015).

As evident from above, most knowledge about involvement of MAPKs during plant defense comes from studies in *A. thaliana* and other dicots, while it is quite limited in important monocot crops. In barley (*Hordeum vulgare*), which is a staple crop for several central-northern European countries, only few studies have addressed the MAPK complement (Křenek et al., 2015; Cui et al., 2019; Li et al., 2019). Barley exhibits both PTI and ETI (Hückelhoven and Seidl, 2016), where the involvement of MAPKs has not been studied before. Based on the previously published results, an array of barley MAPKs strongly respond to *Puccinia hordei* infection, implying their involvement especially in ETI (Křenek et al., 2015).

Plant defense responses are accompanied also by remarkable remodeling of the transcriptome and the proteome. On the proteome level, plant elicitation by flg22 has been associated with the upregulation of LRR-RKs, MAPKs, peroxidases, chitinases, PR proteins, glutathione S-transferases (GSTs), but also proteins linked to membrane transport (Meng et al., 2019; Bassal et al., 2020). Flg22 treatment also triggers substantial changes in protein posttranslational modifications, such as redox modification (Liu et al., 2015), S-nitrosylation (Lawrence et al., 2020) and phosphorylation (Rayapuram et al., 2014). Genetic manipulation of MAPKs remarkably alters proteomes and phosphoproteomes of control and flg22-treated plants (Lassowskat et al., 2014; Takáč et al., 2014, 2016; Rayapuram et al., 2018). These studies, mostly conducted on model plant species, helped to identify proteins regulated by specific MAPK isoforms, including their reversible phosphorylation. Nevertheless, no data on proteome-wide effects of flg22 on barley lines with genetically manipulated MAPK are available so far.

Here, we have used TAL (transcription activator-like) effector nuclease (TALEN) technology to prepare *HvMPK3* knock-out (KO) lines of *H. vulgare* and compared them to the wild type in order to monitor proteome responses during PTI triggered by flg22 application. Comparative proteomic analysis revealed differences in early responses of PR proteins including chitinase 2 and proteins belonging to thaumatin family. This knock-out mutants in *HvMPK3* exhibited also mild root hair phenotypic differences compared to the wild type but more pronounced differences when elicited with flg22.

## Materials and Methods

### Molecular Cloning, Transformation of Zygotic Embryos, and Selection of Transgenic Plants

#### Plant Material and Cultivation

Immature zygotic embryos of spring barley (*Hordeum vulgare*, variety Golden Promise) were used for the preparation of all stably transformed transgenic and control wild type barley lines. Independent T3 and T4 generation barley lines with Z1 TALEN mediated knock-out of the *HvMPK3* gene and non-transformed wild type lines were used for the experiments. In the transgenic lines, designated *HvMPK3* KO-A, *HvMPK3* KO-B and *HvMPK3* KO-C, different homozygous knock-out mutations in the first exon of the *HvMPK3* gene are present and the *Z1 TALEN* gene pair T-DNA cassette is segregated out. Control wild type lines derived from wild type immature zygotic embryo culture were designated WT-A, WT-B, and WT-D. Immature zygotic embryo donor plants were grown in phytotron (Weiss-Gallenkamp, Loughborough, United Kingdom) at 15°C for 16 h in the light (day), 12°C for 8 h in darkness (night), 70% relative humidity with light levels of 450–500 μmol.m^–2^.s^–1^. Illumination was provided by cool white fluorescent tubes (Philips Master tl-d 58W/840) supplemented with clear incandescent light bulbs (Crompton 40W Cooker Hood Lamp). Donor plants were grown in the 3:1 mixture of 10956 Topf+TonL+Perl professional substrate (Gramoflor, Germany) and PF12100112 professional substrate (Rašelina Soběslav, Czechia) supplemented with additional perlite and fed several times with CERERIT GSH (Lovochemie, Czechia) during vegetation. Transgenic and control lines were propagated in the same substrate mixture under similar growing conditions in phytotron, or in an air-conditioned containment greenhouse under natural light conditions, supplemented with red-yellow sodium-vapor lamps (Osram Vialox NAV-T Super 4Y 600W).

#### Computational Characterization of the *HvMPK3* Gene

Previously, we identified the annotated *HvMPK3* gene (gene code MLOC_17814), originally designated as *HvMPK5*, in the *H. vulgare* genome assembly of cv. Morex (version: 082214v1^1^) (Křenek et al., 2015). In the present work, we use the designation HvMPK3 to follow nomenclature recently suggested for *Triticeae* MAPK family (Goyal et al., 2018). HvMPK3 is the closest barley homologue of the *Arabidopsis* MPK3. Latest annotated version of the *HvMPK3* gene (gene code HORVU4Hr1G057200), which is present in the current cv. Morex genome assembly (version: IBSC_v2, see text footnote 1; Mascher et al., 2017) was used for analysis in this study.

Aligned splicing variants of the *HvMPK3* gene were downloaded from the transcript comparison view of the Ensembl Plants *HORVU4Hr1G057200* gene model interface. The FASTA format of the amino acid sequence of the HORVU4Hr1G057200.4 was downloaded from the Ensembl Plants interface using “Export data” utility. The FASTA format of the amino acid sequence of *Arabidopsis* MPK3 (NP_190150.1) was retrieved from National Centre for Biotechnology Information (NCBI) web site^2^. Amino acid sequences of HvMPK3 and AtMPK3 were aligned using protein-protein BLAST suite of NCBI^3^. Transcribed portion of the genomic sequence of the *HORVU4Hr1G057200.4 HvMPK3* gene was edited in the ApE plasmid editor^4^ and its structural elements were visualized with the Exon-Intron Graphic Maker^5^.

#### Design and Molecular Cloning of *TALEN* Gene Pair Construct

pBRACT214-p*Ubi*-p*Act1* vector was developed from the binary cereal transformation vector pBRACT214 (Biotechnology Resources for Arable Crop Transformation; BRACT) by replacing SapI-SphI restriction DNA fragment (333 bp) in pBRACT214 with a SapI-SphI DNA expression cassette (1852 bp), prepared by a gene synthesis service (GeneCust, France). The synthetic cassette is driven by the non-coding 5′ region of the rice *ACTIN 1* gene ending immediately upstream of the *ACTIN 1* start codon (p*Act1*) (McElroy et al., 1990, 1991) and a nopaline synthase gene (*Nos*) terminator and contains multiple cloning site in between these two transcription regulatory elements. Two expression cassettes are present in pBRACT214-p*Ubi*-p*Act1*, in addition to p*Act1* driven cassette also the original maize ubiquitin promoter (p*Ubi*) driven Gateway^TM^ cassette, allowing for the constitutive co-expression of two different genes or a pair of *TALEN* genes from a single vector backbone in cereals.

Transcription activator-like effector nuclease was designed using TAL Effector Nucleotide Targeter 2.0^6^ (Cermak et al., 2011; Doyle et al., 2012), assembled using the Golden Gate Cloning system (Cermak et al., 2011), and cloned into the ELD/KKR backbone plasmid as described previously (Kasparek et al., 2014). TALEN pair, named Z1F/Z1R or Z1 TALEN pair was designed to recognize following sequences in the first exon of the *HvMPK3* gene (TALEN binding sites underlined): 5′-CCTCTACAACATATTcggcaaccagttcgagaTCACGGCCAAGT ACC-3′. *Z1F TALEN* gene (3 kb) was excised from ELD/KKR-*Z1F* using double digestion with NheI and XbaI. The resulting restriction fragments were dephosphorylated, end-filled and A-tailed with DreamTaq DNA polymerase (Thermo Fisher Scientific, Waltham, Massachusetts, United States) and ligated into the pCR^TM^8/GW/TOPO^®^ vector (Thermo Fisher Scientific) to generate pCR8/GW/TOPO-*Z1F* construct. This was recombined with the destination vector pBRACT214-p*Ubi*-p*Act1* by Gateway^TM^ LR Clonase^TM^ II (Thermo Fisher Scientific) reaction to clone Z1F behind the *pUbi* promoter and produce pBRACT214-p*Ubi:Z1F*-p*Act1* construct. *Z1R TALEN* gene (3 kb) was excised from ELD/KKR-*Z1R* using double digestion with NheI and NotI. Also, to facilitate cloning of *Z1R* behind the *pAct1* promoter, Acc65I-NheI and NotI-SbfI sticky end linkers were generated by annealing of the oligonucleotides Link1_F with Link1_R and Link2_F with Link2_R, respectively (Supplementary Table 1). NheI-NotI restriction fragments of ELD/KKR-*Z1R* and both linkers were ligated to pBRACT214-p*Ubi:Z1F*-p*Act1*, which was double digested with Acc65I and SbfI in multiple cloning site behind the p*Act1* promoter, to generate final *Z1 TALEN* gene pair construct pBRACT214-p*Ubi:Z1F*-p*Act1:Z1R*. All cloning intermediates and final *TALEN* gene pair construct were selected and verified based on the restriction digestion analysis of individual clones obtained after each cloning step. All the diagnostic restrictions involved Kpn2I, which cuts in *HD* repeats of DNA binding domain of *TALENs* producing specific restriction pattern for each *TALEN* gene. Verified *TALEN* gene pair constructs were electroporated into *Agrobacterium tumefaciens* strain AGL1 together with helper plasmid pSOUP (Bartlett et al., 2008).

#### Development of the Transgenic and Control Wild Type Barley Lines

To prepare stable transformed transgenic barley lines, we followed *Agrobacterium*-mediated barley transformation protocol described by Bartlett et al. (2008) and Harwood et al. (2009), with the exception, that 30 mg.l^–1^ hygromycin was used in the plant selection media. Regenerants derived from the same calli can originate from single or multiple transformations. To safely identify independent transgenic lines, regenerants derived from different calli were considered as independent lines, whereas regenerants derived from the same calli were considered as representatives of the same line. Transgenic plants derived from the same calli were considered as an independent lines only if they contained different TALEN-induced homozygous mutations in the *HvMPK3* gene. Control independent wild type barley lines WT-A, WT-B and WT-D were derived from non-transformed zygotic embryos.

#### *TALEN* Gene Pair T-DNA and Mutation Genotyping

Genomic DNA was isolated using the cetyltrimethylammonium bromide based method. To determine presence of the *Z1 TALEN* gene pair T-DNA cassette, a 917 bp DNA fragment of the *hpt* gene was amplified from a genomic DNA of hygromycin resistant plants and their progenies using polymerase chain reaction (PCR) with primer pair hptF/hptR (Supplementary Table 1). Z1 TALEN-induced mutations in the target region of the *HvMPK3* gene were determined by two-step procedure. First, a 380 bp DNA fragment covering the target region of Z1 TALEN pair was amplified from the genomics DNA of transgenic plants using PCR with primers K3F1 and K3R1 (Supplementary Table 1). Second, the 380 bp PCR product was analyzed for Z1 induced mutations by restriction digestion with BsrI (PCR-RE analysis) and/or by direct commercial Sanger sequencing involving *K3F1* primer in the sequencing reaction (SEQme, Czechia). Individual transgenic plants were considered as plants without T-DNA, if they were simultaneously associated with the negative PCR result for the *hpt* gene and with the positive PCR result for the 380 bp *HvMPK3* DNA fragment.

### RT-qPCR Quantification of the *HvMPK3* Gene Expression

Barley seeds were surface sterilized in 70% (v/v) ethanol for 2 min followed by incubation in 5% (v/v) sodium hypochlorite supplemented with 100 μl of Tween-20 for 8 min upon shaking. After thorough washing in sterile DW (5 times), seeds were incubated in sterile DW at 4° C overnight and subsequently placed on solid Fåhreus medium with nitrogen (FAH) (Fahraeus, 1957) in square Petri dishes. Following seed stratification for 3 days at 4°C, Petri dishes with seeds were placed vertically on single shelf in phytotron (Weiss-Gallenkamp) and incubated at 21°C, 16/8 h (light/dark) photoperiod, 70% relative humidity with light levels of 140–150 μmol.m^–2^.s^–1^ provided by cool white fluorescent tubes (Philips Master tl-d 58W/840). Before treatment, 4 days old seedlings were pre-cultivated in glass Petri dishes (145 mm in diameter) filled with 50 ml of liquid FAH medium for 24 h. In each Petri dish, four seedlings of the same line were pre-cultivated. During pre-cultivation, Petri dishes with seedlings were gently swayed on MR-12 Rocker-Shakers (Biosan, Riga, Latvia), which were placed in the phytotron. After 24 h of pre-cultivation, liquid FAH medium in Petri dishes was carefully replaced with 50 ml of fresh liquid FAH medium supplemented with flg22 at 1 μM final concentration (flg22 treatment) or with 50 ml of fresh liquid FAH medium (mock treatment). Petri dishes (each with four seedlings) were placed back on MR-12 Rocker-Shakers immediately following medium exchange. Cultivation conditions were the same as for growing of seedlings on solid FAH medium. The only difference was in light level, which was decreased to 40–50 μmol.m^–2^.s^–1^. Root samples for the RT-qPCR analysis were collected after 6 h of seedling cultivation in mock or flg22 conditions. Samples from two plants of the same genotype/treatment combination were pooled and two biological replicates of pooled samples were analyzed per each genotype/treatment combination within the single experiment. RNA extraction, cDNA preparation and qPCR analysis including data evaluation were essentially performed as described before (Křenek and Smékalová, 2014; Křenek et al., 2015) and involved two technical replicates in qPCR analysis. The whole RT-qPCR experiment was repeated two times and in total four biological and eight technical replicates were analyzed.

### Preparation of the Plant Material for Phenotypic Analyses

Two independent barley *HvMPK3* KO lines (B, C) and wild type (WT A) caryopses were plated on solid ½MS medium and allowed to stratify for 2–3 days at 4°C. Subsequently, petri dishes were transferred to phytochamber and cultivated under the conditions described above. Grown seedlings were harvested between 3 and 5 days after germination. For examination of flg22 effects on root morphology, 3 days old seedlings were transferred to solid ½MS medium supplemented with 1 μM flg22 (Cambridge Research Biochemicals, Billingham, United Kingdom) for 1–6 days.

### Visual Documentation and Microscopy of Wild Type and HvMPK3 KO Root Hair Phenotypes

Root hair phenotypes were addressed in more detail using the stereo zoom microscope AxioZoom.V16 (Zeiss, Oberkochen, Germany) and when necessary, roots were also documented with differential interference optics (DIC) of an upright AxioImager M2 widefield microscope (Zeiss, Oberkochen, Germany). Root hair measurements were done on terminally elongated root hairs of control or flg22-treated wild type and *HvMPK3* KO roots. In each case, a total of more than 100 root hairs from 8 to 9 roots were taken into account in one biological replicate. All measurements derived from 3 biological replicates (in total 25 roots and more than 400 root hairs per case). In all cases, statistical comparisons were done pairwise with Student’s *t*-test.

### Differential Shot-Gun Proteomic Analysis

Within this study, two experiments were conducted for proteomic analyses. First, the proteomes of 5 days old *HvMPK3* KO plants lines A, B and C (three plants per line) were compared to the wild type (lines A, B and D). In the second, the same lines were exposed to liquid ½ MS medium with or without 200 nM flg22 for 6 h. Roots (experiment 1 and 2) and shoots (experiment 1) of three plants for each line and treatment variant were pooled for one sample. Plant material was homogenized in liquid nitrogen to fine powder and proteins were extracted using phenol extraction and methanol/ammonium acetate precipitation, as described previously (Takáč et al., 2017). Together 50 μg of proteins dissolved in 50 μl of 6 M urea were subjected to in solution trypsin digestion.

Prior to trypsin application, urea extracts were supplemented with 10 μl of 50 mM dithiothreitol and incubated at room temperature for 1 h to reduce the disulphide bonds. Next, reduced thiol groups were alkylated by addition of 10 μl of 50 mM iodoacetamide, and reaction mixture was incubated at room temperature for 1 h. Afterward, the urea concentration in extracts was lowered to less than 1 M by HPLC grade water to avoid trypsin inhibition. The trypsin digestion (1 μg of sequencing grade modified trypsin from Promega per 50 μg of proteins) was carried out by gentle shaking at 37°C overnight. Trypsin digestion was stopped by 4 μl of concentrated acetic acid and the peptides were cleaned on C18 cartridges (Bond Elut C18; Agilent Technologies, Santa Clara, CA) according to manufacturer’s instructions. Peptides eluted by 90% (v/v) acetonitrile were dried using SpeedVac and used for nLC-MSMS.

#### Nano-Liquid Chromatography-Tandem Mass Spectrometry Analysis (nLC-MSMS)

Two micrograms of protein tryptic digest were subjected to nLC-MSMS analysis as published previously (Takáč et al., 2016). Briefly, peptides were separated using reversed phase C18 75 μm × 150 mm column and Ultimate 3000 HPLC system (both Thermo Fisher Scientific) via nonlinear, 170 min long, constant flow (0.3 μl min^–1^) gradient of acetonitrile (in 0.1% (v/v) formic acid) as follows: 2–55% (v/v) for 125 min, 95% (v/v) for 20 min, 2% (v/v) for 25 min.

Mass spectra were collected using the linear trap detector of the nano-electro spray ionization LTQ-Orbitrap Velos mass spectrometer (Thermo Fisher Scientific) directly linked to the nLC system. The mass spectrometer operated in the data dependent acquisition (DDA) mode of 18 scan events: one MS scan (m/z range: 300–1700) followed by 17 MSMS scans for the 17 most intense ions detected in MS scan. Other parameters included Spray voltage: 1.95 kV, Capillary temperature: 237°C, S-Lens RF Level: 65%; Automatic Gain Control “On” with AGC target settings of 1.00e^+4^, and Maximum inject time of 50 ms for both Full MS and MSn; number of microscans: 1 for both MS1 and MS2 scans; Dynamic Exclusions allowed with Repeat count: 1, Repeat Duration: 30.0 s, Exclusion list size: 500, Exclusion duration: 180 s; Activation type: CID, Default charge state: 2, Isolation width: 2.0 m/z, Normalized collision energy: 35, Activation Q: 0.250, Activation time: 30 ms. All sample files were deposited to publicly accessible database (see Data Availability Statement for details).

#### Protein Identification and Relative Quantification

The raw data files were searched using the SEQUEST algorithm of the Proteome Discoverer software version 2.1 (Thermo Fisher Scientific), as described previously (Takáč et al., 2017). Variable modifications were considered for: cysteine carbamidomethylation (+57.021), methionine oxidation (+15.995), methionine dioxidation (+31.990), and phosphorylation (+79.966) of serine, tyrosine, and threonine. Maximum three modifications were allowed per peptide. Two missed cleavage sites were permitted, while precursor (MS1) and fragment ion (MS2) tolerances were set to 1.8 and 0.8 Da, respectively. To compensate for the large MS1 mass tolerance aimed to include isotopic precursor peaks, the decoy database searching and tight FDR control were implemented to obtain confident results. *H. vulgare* NCBI protein database (as of August 2019, with 25,395 entries) served as the target database, while its reversed copy (created automatically by the software) served as a decoy database. The proteins without functional annotations were searched against the UniProt SwissProt database (release 07/2019) using BLAST+. The function of the best blast hit was used to annotate the protein. Only high confidence protein identifications (FDR < 1%) were further considered. Identified proteins were grouped by default parameters of the software, defining the group as proteins strictly necessary to explain presence of identified peptides. A representative/master protein of the group is the protein with highest score, spectral count and number of matched peptides. If those parameters are equal, the protein with longest sequence is designated as a master protein. The proteins presented in results are all master proteins. For the relative quantitative analysis, an in-house script was developed (see Supplementary Material). It utilizes results.xlsx files exported by the Proteome Discoverer software. The quantitation is based on sums of precursor ion intensities of peptides attributed to particular proteins. Results were normalized by factors that were calculated to equalize total ion intensities of all Peptide Spectral Matches across biological samples, and their respective replicates (n = 3). Normalized average protein intensities (value from one line was considered as a replicate) were used to calculate fold changes when comparing biological samples. Maximum of five most intensive precursor ions per protein were considered. The ANOVA *p* ≤ 0.05 was used to filter statistically significant results, applied to proteins exhibiting the fold change ≥ 1.5.

Proteins identified by 1 peptide were excluded from results. Proteins present in all three lines corresponding to the control proteome and absent in all three lines of the test proteome were considered as unique for the control proteome, and vice versa.

#### Bioinformatic Proteome Analysis

The differential proteome was evaluated using gene ontology (GO) annotation and KEGG (Kyoto encyclopedia of genes and genomes) pathways analyses, as well as protein domains identification using the Functional analysis module of OmicsBox software (BioBam Bioinformatics, Valencia, Spain). Sequences in FASTA format were blasted using *H. vulgare* database, allowing 3 blast hits per sequence. Proteins were annotated using GO weight 5 against green plants database. The output was simplified by GO Slim function. STRING (Szklarczyk et al., 2015) application was used for projection of protein interaction network applying minimum required interaction score 0.55.

Amino acid sequences of differentially abundant proteins in wild type as well as KO lines in response to flg22 were screened for the presence of MAPK specific docking domains using Eukaryotic Linear Motif (ELM) resource. The identified proteins were further screened for the presence of MAPK-specific phosphorylation motif by using GPS 3.0–Kinase-specific Phosphorylation Site Prediction.

### Western Blot Analysis

The MAPK activation was examined in roots of wild type and *HvMPK3* KO plants treated with 200 nM flg22 as described for RT-qPCR analysis for the time points indicated in the respective figure. For HSP70 abundance analysis, roots of 5 days old seedlings of WT and *HvMPK3* KO lines were harvested.

Roots were flash frozen in liquid nitrogen and ground to fine powder in precooled mortar and pestle. Proteins were extracted as described before (Takáč et al., 2017). Separated proteins were then transferred on polyvinylidene difluoride (PVDF) membranes overnight at 24V. Subsequently, transfer was verified by Ponceau S staining of the membranes, which were then blocked in 5% (w/v) bovine serum albumin (BSA) in Tris-buffered saline supplemented with 0.1% (v/v) Tween 20 (TBS-T) overnight at 4°C. Blocked membranes were incubated with primary anti-HSP70 antibody (Agrisera, Sweden), diluted 1:5000 and with primary polyclonal antibody against mammalian phosphorylated ERK1/2 (phospho-p44/42 (pERK); Cell Signaling 9101) diluted 1:1000 in 1% (w/v) BSA in TBS-T, overnight at 4°C. Membranes were washed thoroughly and subsequently incubated for 1.5 h at room temperature with HRP-conjugated secondary antibody (Thermo Fisher Scientific), diluted 1:5000 in 1% (w/v) BSA in TBS-T. Following three washing steps, PVDF membranes were incubated with commercial Clarity Western ECL Substrate (BioRad, Hercules, CA, United States) and documented in a ChemiDoc MP imaging system (BioRad). After protein detection using polyclonal pERK antibody, the membrane was washed in TBS-T and incubated three times in 0.5 M NaOH for 20 min to wash out the primary antibody. After four washing steps in MilliQ water (10 min each) and three washing steps using TBS-T (15 min each), the membrane was blocked in 5% (w/v) BSA supplied in TBS-T, at 4°C overnight. Afterward, the membrane was incubated with monoclonal pERK antibody (phospho-p44/42; Cell Signaling 5726), diluted 1:750 in 1% (w/v) BSA in TBS-T, at 4°C overnight. Next steps were identical to the protocol used for polyclonal pERK antibody.

The band densities were quantified using ImageJ software. All immunoblot analyses were performed in three biological replicates. Student’s *t*-test was applied to evaluate the statistical significance of differences.

### Analysis of Chitinase Activity

Chitinase activity was examined in native extracts from plants treated as for proteomic analyses and visualized on native PAGE gels complemented with 1% (w/v) glycol chitin as described in Békésiová et al. (2008). The band densities were quantified using ImageJ software. Chitinase activity analyses were performed in three biological replicates. Student’s *t*-test was applied to evaluate the statistical significance of differences.

## Results

### Generation and Selection of TALEN Knock-Out Lines of *HvMPK3*

**FIGURE 1:**
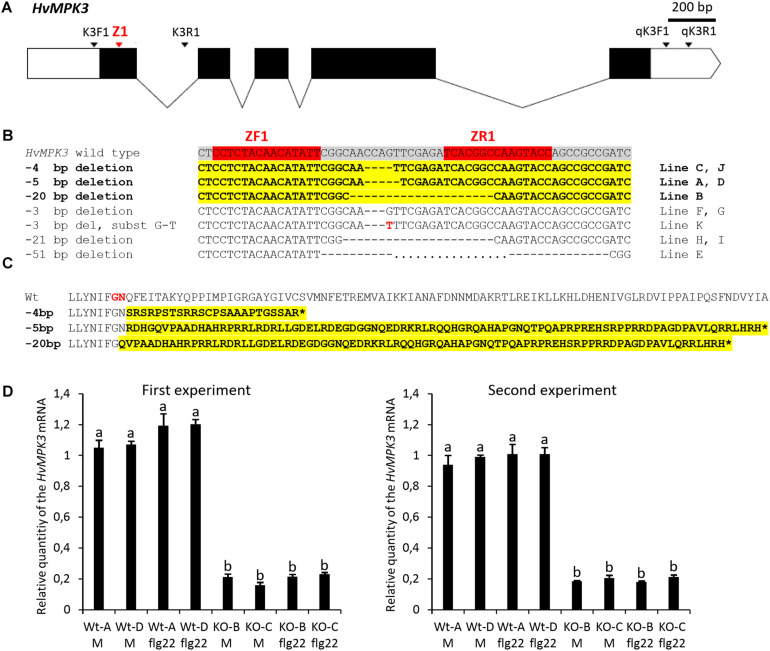
Development and characterization of the homozygous *HvMPK3* knock-out (KO) barley lines. **(A)** Schematic representation of the transcribed portion of the *HvMPK3* gene (HORVU4Hr1G057200, Ensembl Plants, http://plants.ensembl.org/index.html). HORVU4Hr1G057200.4 splicing variant coding for the 369 amino acid long HvMPK3 protein is depicted. Coding exons are shown as black rectangles and 5′ and 3′ untranslated regions are shown as open rectangles. Introns are indicated by solid lines. Binding sites of the Z1 TALEN pair are indicated by red arrowhead in the coding region of the first exon (Ex1). Annealing positions of the K3F1/K3R1 primers (mutation genotyping) and of the qK3F1/qK3R1 primers (RT-qPCR) are indicated by black arrowheads. **(B)** Z1 TALEN pair-induced mutations observed in 11 independent lines in T1 generation. Red boxes in the wild type *HvMKP3* sequence indicate binding sites of the ZF1 and ZR1 monomers of the Z1 TALEN pair. Black dashes indicate the identified deletions. The size of the deletion is shown on the left of each mutated sequence. Transgenic lines indicated on the right of each mutated sequence were homozygous for the respective mutations in the T1 generation. Sequences harboring frame-shift (loss-of-function) mutations are shown in bolt and highlighted in yellow. Also, the respective designations of the deletion sizes and lines are shown in bolt. **(C)** Putative truncated versions of the HvMPK3 protein associated with the Z1 TALEN pair-induced loss-of-function mutations. Reference wild type HORVU4Hr1G057200.4 HvMPK3 protein and its truncated versions are translated from the nucleotide sequences starting two base pair upstream of the ZR1 TALEN binding site. The size of the respective loss-of-function deletion is shown on the left of each truncated HvMPK3 protein. Aberrant peptide sequences resulting from frameshift translations are shown in bolt and highlighted in yellow. The frameshift occurs after decoding of the 26th codon (–20 bp deletion) or 27th codon (–4 and –5 bp deletions) in the HORVU4Hr1G057200.4 *HvMPK3* gene. Amino acids coded by the 26th codon and 27th codon of the gene are shown in red in the wild-type *HvMPK3* sequence. Translation termination at premature stop codon is indicated by asterisk. **(D)** Relative quantity of the *HvMPK3* mRNA in the roots of the *HvMPK3* KO and control barley lines. Four days old intact seedlings of the *HvMPK3* KO lines *HvMPK3* KO-B (KO-B) and *HvMPK3* KO-C (KO-C) and wild type control lines A (WT-A) and D (WT-D) were incubated in liquid Fåhreus medium with nitrogen (FAH) (M – mock treatment) or in liquid FAH medium supplemented with 1 μM flg22 (flg22 – flg22 treatment) in two biological replicates for 6 h. The expression of the *HvMPK3* gene was normalized to the expression of the reference *HvMPK14* gene and is shown as relative to the single biological replicate of the WT-A Mock sample. An average value of two biological replicates is plotted per each genotype/treatment combination. Error bars indicate standard deviations from two biological replicates. Data were analyzed by one-way ANOVA with the Tukey’s *Post hoc* test. Means with different letters are significantly different at *P* < 0.01. Experiment was repeated two times with similar results (First and Second experiment).

We designed a Z1 TALEN pair to target coding sequence of the first exon of the *HORVU4Hr1G057200.4* splicing variant of the *HvMPK3* gene (Figure 1A and Supplementary Figure 1). There are eight different splicing variants associated with the annotated *HvMPK3* gene (*HORVU4Hr1G057200*, see text footnote 1) and among those, *HORVU4Hr1G057200.4* codes for the longest HvMPK3 protein, which is also the most similar to the *Arabidopsis* MPK3 (Supplementary Figures 1, 2). Specifically, a 369 amino acid long sequence of *HORVU4Hr1G057200.4* covers the 370 amino acid long sequence of the *Arabidopsis* MPK3 with 73% amino acid identity and 87% amino acid similarity (Supplementary Figure 2). Designed Z1 TALEN pair also targets coding sequence of the *HORVU4Hr1G057200.3* splicing variant, which codes for the second longest HvMPK3 protein containing 336 amino acids (Supplementary Figure 1). However, it does not target the coding sequences of the remaining six splicing variants.

Two rounds of transformation of in total 580 immature zygotic embryos of wild type barley with Z1 *TALEN* gene pair construct pBRACT214-p*Ubi:Z1F*-p*Act1:Z1R* resulted in the regeneration of 12 independent hygromycin-resistant barley lines represented by 20 plants. PCR genotyping of T0 and T1 generation plants confirmed the presence of *hygromycin phosphotransferase* (*hpt*) gene, which is a part of the *TALEN* gene pair T-DNA cassette, in 11 independent Z1 transgenic lines (out of 11 Z1 lines genotyped). To identify TALEN-induced mutations in the first exon of the *HvMPK3* gene, a 380 bp DNA fragment covering the binding sites of Z1 TALEN pair was amplified from genomic DNA of transgenic plants using PCR with primers *K3F1/K3R1* (Figure 1A, Supplementary Figure 1, and Supplementary Table 1). Restriction digestion with BsrI and direct sequencing of the 380 bp PCR amplicons resulted in the identification of homozygous mutations in the target region of the *HvMPK3* gene in 11 independent Z1 lines in T1 generation (Figure 1B). All the observed mutations were deletions ranging from 3 to 51 bp in size and in total six different homozygous deletions were found in the genomes of 11 independent Z1 lines. In one case, 3 bp deletion was combined with G-T substitution. We identified five independent lines bearing three distinct homozygous *HvMPK3* knock-out (frame-shift) deletions (Figure 1B). In particular, lines C and J contain −4 bp deletion, lines A and D contain −5 bp deletion and line B contains −20 bp deletion in the target region of the first exon of the *HvMPK3* gene. Also, one line (line E) harbors a large homozygous 51 bp deletion, which results in the depletion of the ATP-binding pocket in the predicted amino acid sequence of the corresponding HvMPK3 mutant protein. Homozygous deletions harbored by lines A (−5 bp), B (−20 bp), C (−4 bp), D (−5 bp), and E (−51 bp) were uniformly transmitted from T1 to T2 generation, suggesting stable inheritance of these mutations. As confirmed by PCR genotyping for the presence of the *hpt* gene, in the T1 and T2 generation, Z1 *TALEN* gene pair T-DNA cassette segregated away from the genomes of the lines A, B and C and also from the genome of the line E. The selected *HvMPK3* knock-out T-DNA free lines, which were analyzed in this work are further designated *HvMPK3* KO-A, *HvMPK3* KO-B and *HvMPK3* KO-C.

Frameshifts in the *HORVU4Hr1G057200.4 HvMPK3* gene caused by −4, −5, and −20 bp deletions are predicted to result in severely truncated abnormal HvMPK3 proteins (Figure 1C). To check if these mutations had an effect on the *HvMPK3* mRNA expression, we analyzed T3 generation seedlings of the *HvMPK3* KO-B, *HvMPK3* KO-C and WT control lines by RT-qPCR (Figure 1D). The relative level of the *HvMPK3* mRNA was approximately 5 to 6 times lower in the roots of *HvMPK3* KO-B and *HvMPK3* KO-C seedlings than in the roots of control seedlings. Primers qK3F1/qK3R1, which were used in the RT-qPCR analysis, allow for nearly overall quantification of the *HvMPK3* gene expression, because they anneal to the 3′ untranslated region of the seven out of eight *HvMPK3* splicing variants (Supplementary Figure 1 and Supplementary Table 1). We targeted by Z1 TALENs coding regions of two splicing *HvMPK3* variants only (Supplementary Figure 1). The observed results may therefore suggest that the induced deletions have negative effect on the mRNA level of all or most of the *HvMPK3* splicing variants. There was no significant difference in the level of *HvMPK3* mRNA between the roots treated with 1 μM flg22 for 6 h and mock treated roots in both knock-out and control lines. The whole experiment was repeated two times with similar results (Figure 1D).

### Activation of HvMPK3 in Response to flg22

**FIGURE 2:**
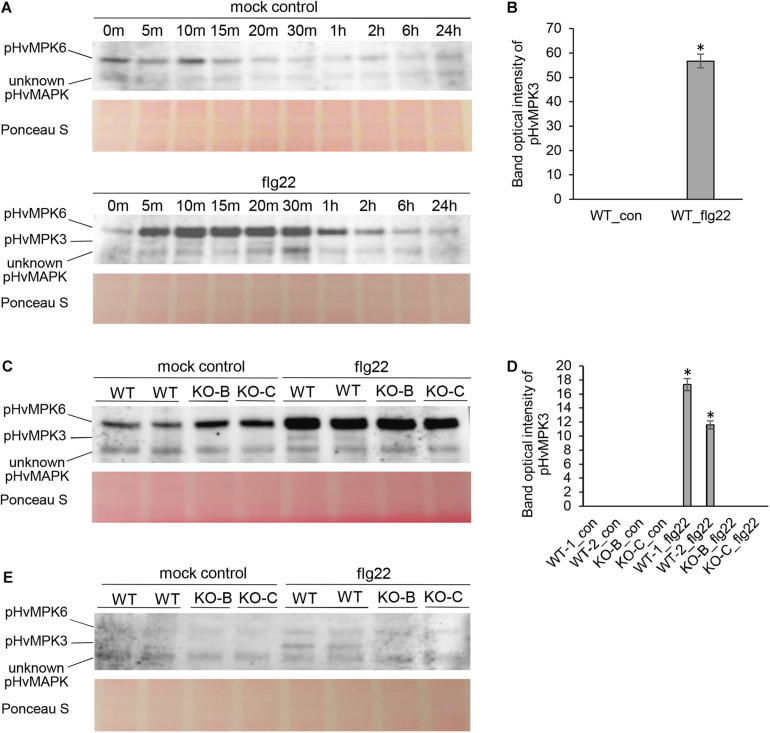
Activation of barley pHvMPK6, pHvMPK3 and unknown pHvMPK in roots of wild type (WT) and *HvMPK3* KO lines (KO-B and KO-C) after flg22 treatment detected by immunoblotting analyses. **(A)** Time course activation of barley MAPKs in flg22-treated roots of WT seedlings as detected using polyclonal anti-pERK antibody. **(B)** Quantification of band intensities in **(A)** after 15 min of treatment. Asterisks indicate statistically significant differences between flg22-treated and mock-treated WT at *p* < 0.05 (Student’s *t*-test). **(C)** Activation of MAPKs in roots of WT and *HvMPK3* KO lines after 15 min long flg22 treatment, as detected by polyclonal anti-pERK antibody. **(D)** Quantification of band intensities in **(C)**. Asterisks indicate statistically significant differences between flg22-treated WT and *HvMPK3* KO lines at *p* < 0.05 (Student’s *t*-test). **(E)** Activation of MAPKs in roots of WT and *HvMPK3* KO lines after 15 min long flg22 treatment, as detected by monoclonal anti-pERK antibody. Uncropped, full original images of the whole immunoblots are provided in Supplementary Figures 6, 7.

In order to investigate the activation of HvMPK3 in response to flg22, we implemented immunoblotting analysis with commercial polyclonal anti-pERK (p44/42) antibody (Figures 2A–D). It detects phosphorylated pMPK6, pMPK4, and pMPK3 in *Arabidopsis* (Frei dit Frey et al., 2014; Smékalová et al., 2014; Samakovli et al., 2020). Flg22 treatment lead to appearance of 3 bands immunoreactive to polyclonal anti-pERK (p44/42) antibody. By alignment to the published pattern of *Arabidopsis* pMPK6 and pMPK3 obtained by the same antibody, and predicted molecular weight of HvMPK6 (44.27 kDa) and HvMPK3 (42.82 kDa), we annotated the upper band as HvMPK6 and intermediate band as HvMPK3 while the identity of lower band remains unknown. Our analysis confirmed the activation of pHvMPK6 and pHvMPK3 in response to flg22. We observed the strongest activation of pHvMPK3 after 10, 15 and 20 min in WT plants, while no HvMPK3 activation was found in mock-treated WT plants (Figures 2A,B). Next, we compared the activation of MAPKs in WT and *HvMPK3* KO plants after 15 min treatment with flg22. This analysis showed phosphorylation of pHvMPK6 and an unknown pHvMPK in *HvMPK3* KO plants which was comparable to WT, while the band corresponding to pHvMPK3 was not present in *HvMPK3* KO plants (Figures 2C,D). Similar result was obtained by using monoclonal pERK (p44/42) antibody which recognized a prominent pHvMPK3 protein band in WT plants but not in *HvMPK3* KO lines (Figure 2E). In conclusion, HvMPK3 is activated by phosphorylation in response to flg22.

### Constitutive Proteome of *HvMPK3* KO Mutants as Compared to Wild Type

To investigate the impact of *HvMPK3* knock-out on barley proteome, we performed a comparative MS-based proteomic analysis of *HvMPK3* KO lines and wild types. First, we compared the root and shoot proteomes of the three *HvMPK3* KO lines with the three wild types under control conditions.

**FIGURE 3:**
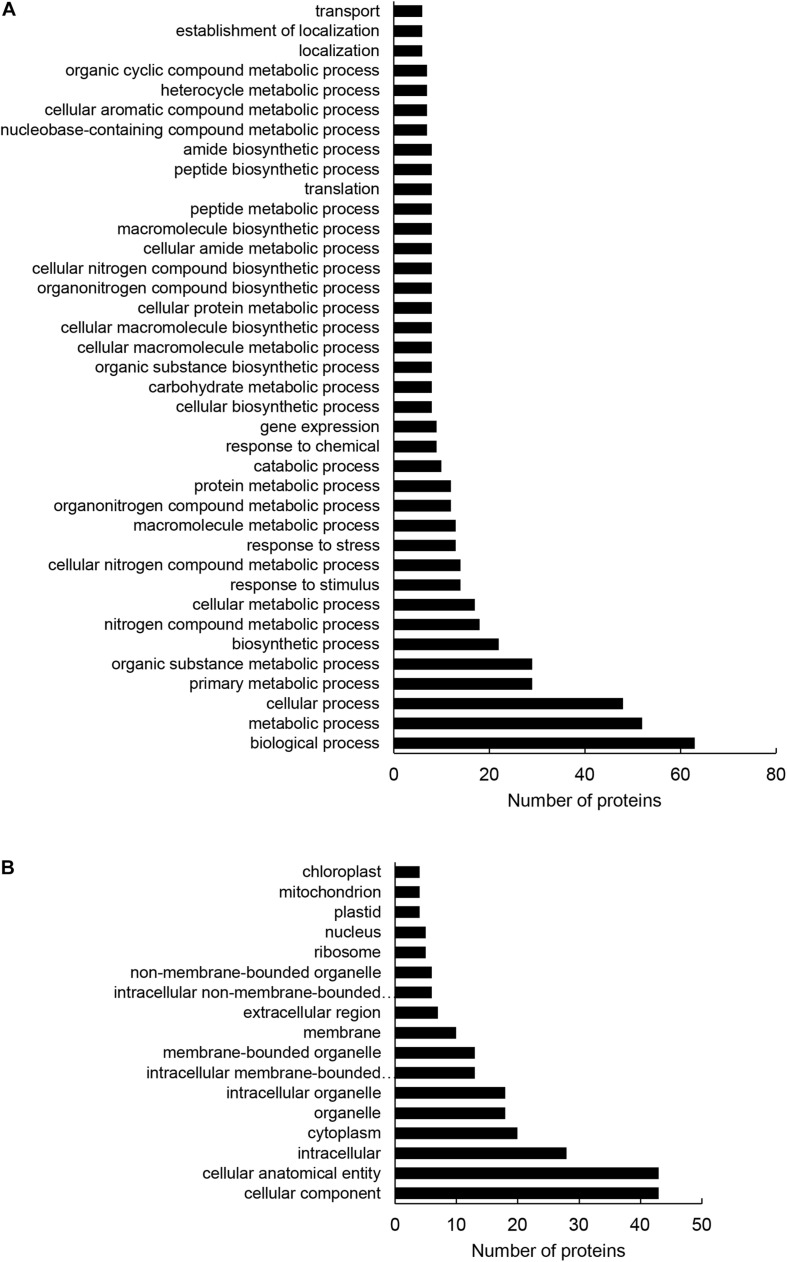
Gene ontology (GO) annotation analysis of root differentially abundant proteins found between *HvMPK3* KO lines and wild types. **(A,B)** GO annotation according to biological process **(A)** and cell compartment **(B)**.

**FIGURE 4:**
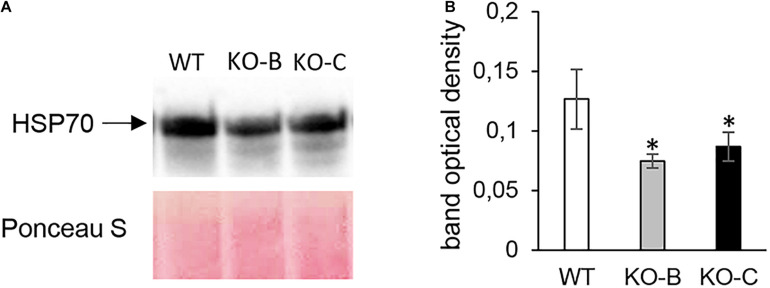
Immunoblotting analysis of HSP70 abundance in barley *HvMPK3* KO lines. **(A)** Immunoblot showing the abundance of HSP70 in roots of wild type (WT) as well as two independent *HvMPK3* KO lines (KO-B and KO-C). **(B)** Quantification of band intensities in **(A)**. Asterisks indicate statistically significant differences between WT and *HvMPK3* KO lines; ^∗^*p* < 0.05, Student’s *t*-test. Uncropped, full original image of the immunoblot is documented in Supplementary Figure 8.

Altogether, 97 differentially abundant proteins in the *HvMPK3* KO lines were found when compared to the wild type, of which 46 were downregulated while 51 showed increased abundances (Supplementary Data Sheet 1A). We classified the obtained differential proteome using GO annotation analysis showing that proteins involved in metabolic processes, response to stress and gene expression were affected in the *HvMPK3* KO lines (Figure 3A and Supplementary Data Sheet 1B). Differential proteome consisted mainly of proteins localized in the cytoplasm, membranes and extracellular space (Figure 3B and Supplementary Data Sheet 1C). Regarding metabolic pathways, purine, sucrose and starch, pyruvate, galactose and amino acid (alanine, aspartate, and glutamate) metabolism were affected, as shown by KEGG pathway analysis (Supplementary Figure 3 and Supplementary Data Sheet 1D). Next, we focused on proteins classified into GO annotation called response to stress. Out of 13 proteins in this annotation, 9 were downregulated (Supplementary Data Sheet 1B). More detailed examination of proteins allowed to differentiate between abiotic and biotic stress-related proteins (Supplementary Data Sheet 1A). We have noticed a downregulation of several abiotic stress-related proteins such as two glutathione S-transferases, germin-like protein, desiccation protectant protein Lea14 homolog and SRC2 (soybean gene regulated by cold-2) homolog. Two annexin domain-containing proteins were found to have increased abundances in the *HvMPK3* KO lines. Moreover, heat shock cognate 70 kDa protein showed downregulation in the KO mutants, which was further validated by immunoblotting analysis (Figures 4A,B).

**FIGURE 5:**
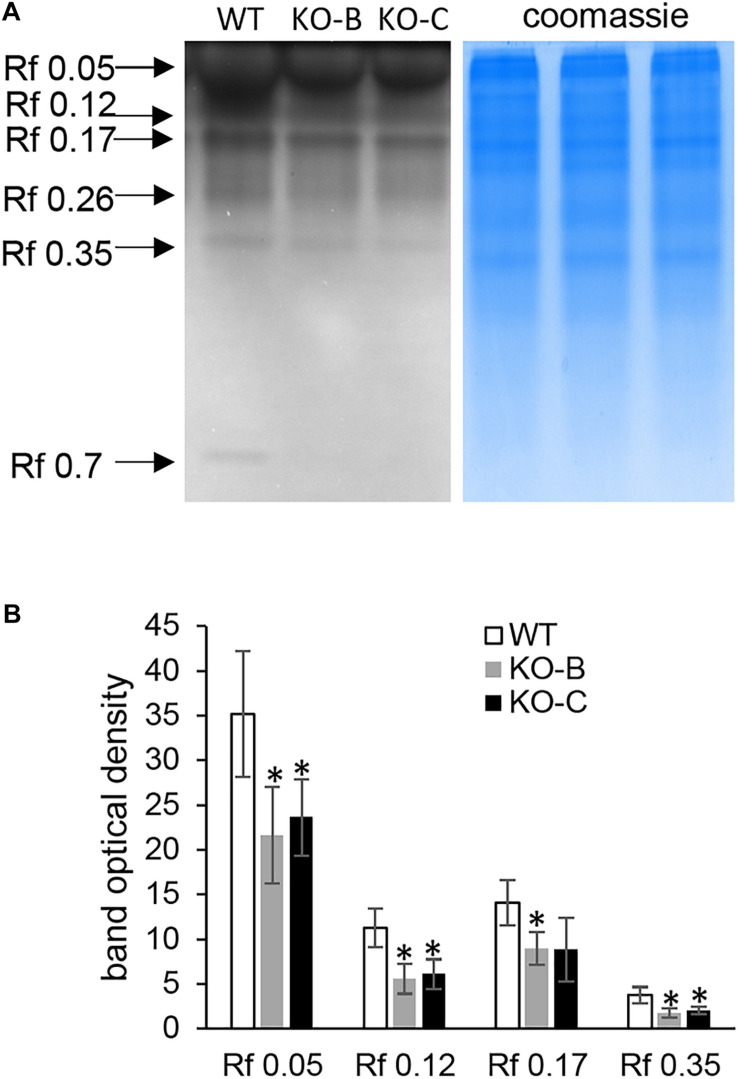
Examination of chitinase activity in roots of wild type (WT) and *HvMPK3* KO lines (KO-B and KO-C). **(A)** chitinase activity on native PAGE gels. Arrows indicate chitinase isozymes with designated relative mobility (Rf). Right panel: visualization of proteins on gel in A by Coomassie staining. **(B)** Quantification of band intensities in **(A)**. Asterisks indicate statistically significant differences between WT and *HvMPK3* KO lines; ^∗^*p* < 0.05, Student’s *t*-test. Uncropped, full original image of the gel is documented in Supplementary Figure 9A.

In total, five proteins were identified bearing secretory peroxidase domain (Supplementary Data Sheet 1E), out of which four exhibited reduced abundance in the KO lines (Table 1 and Supplementary Data Sheet 1A). Secretory peroxidases are known as proteins with defense-related functions during pathogen attack (Johrde and Schweizer, 2008). This is further supported by downregulation of PR proteins, such as thaumatin-like protein TLP8, thionin THI2 and two chitinase isoforms (Table 1). Next, we examined a native chitinase activity in the analyzed KO lines, by visualization on native polyacrylamide gels. These data positively correlated with proteomics results, since chitinase activity was significantly lower in the KO mutants compared to the wild type (Figure 5). On the other hand, another PR protein, pathogenesis-related protein PRMS together with 23 kDa jasmonate-induced protein, showed increased abundances in the KO mutants (Table 1).

**TABLE 1:** Differentially abundant defense related proteins found in *HvMPK3* KO mutant as compared to wild type.

Accession	MW (kDa)	calc. pI	Description	Ratio (*HvMPK3* KO vs. WT)	*P*-value
326496709	36.7	8.06	PER12 Peroxidase 12 (*Arabidopsis thaliana*)	0.23	0.01
326502638	34.1	9.39	PER52 Peroxidase 52 (*Arabidopsis thaliana*)	0.65	0.02
326495784	35.5	7.9	PER12 Peroxidase 12 (*Arabidopsis thaliana*)	0.51	0.02
326494942	34.8	6.55	PER35 Peroxidase 35 (*Arabidopsis thaliana*)	0.45	0.04
326529001	38.6	6.32	Sb03g046810 Cationic peroxidase SPC4 (*Sorghum bicolor*)	4.24	0.001
326489434	44.7	5.67	CLP Chitinase CLP (*Oryza sativa* subsp. *japonica*)	0.24	0.01
326508800	32	5.48	Chitinase 2 (*Tulipa saxatilis* subsp. *bakeri*)	0.49	0.03
112821174	22.9	6.38	23 kDa jasmonate-induced protein (*Hordeum vulgare*)	1.53	0.03
326529301	18.7	9.11	PRMS Pathogenesis-related protein PRMS (*Zea mays*)	3.17	0.05
14164983	24.3	7.56	thaumatin-like protein TLP8 (Hordeum vulgare)	Unique in WT	NA
326506728	14.5	5.81	THI2 Thionin (*Brassica rapa* subsp. *pekinensis*)	Unique in WT	NA
326491885	36.1	6.43	RACK1A Guanine nucleotide-binding protein subunit beta-like protein A (*Oryza sativa* subsp. *japonica*)	1.55	0.01
326502418	43.1	5.66	OLE9 Glucan endo-1,3-beta-D-glucosidase (*Olea europaea*)	Unique in KO	NA

Furthermore, mutant KO lines show deregulation of proteins involved in membrane transport and villin2, an actin binding protein. Two histone isoforms and translation-related proteins also showed alterations in their abundances (Supplementary Data Sheet 1A).

Very similar differences between proteomes of the wild type and mutant KO lines were found in aerial plant parts (Supplementary Data Sheets 2A–E and Supplementary Figure 4). Mutants showed reduced abundances of chitin elicitor-binding protein and two peroxidase isoforms. Moreover, several abiotic stress-related proteins, including annexin D2 and Cu/Zn superoxide dismutase were downregulated.

We also interrogated the mass spectrometry data for S,Y,T phosphorylation in the proteomes of untreated roots and shoots of WT and *HvMPK3* KO lines. Six reproducibly appearing phosphopeptides were identified in the roots of WT belonging to five proteins (Supplementary Data Sheet 3). These included peroxidase 52, actin 1, phosphoglycerate kinase, fructose-bisphosphate aldolase 5 and MICOS complex subunit MIC60. Remarkably, 4 phosphopeptides were found in the root proteome of *HvMPK3* KO lines, and all of them belonged to ACTIN 1, indicating its multiple phosphorylation. The phosphopeptide profiles of shoots did not differ substantially between WT and *HvMPK3* KO line (Supplementary Data Sheet 3).

These results indicate that proteins related to biotic and abiotic stresses are constitutively downregulated in *HvMPK3* KO lines. Therefore, we focused next experiments on the responses of *HvMPK3* KO lines to the bacterial elicitor flg22.

### *HvMPK3* Knock-Out in Barley Leads to Proteome Remodeling in Response to flg22

**FIGURE 6:**
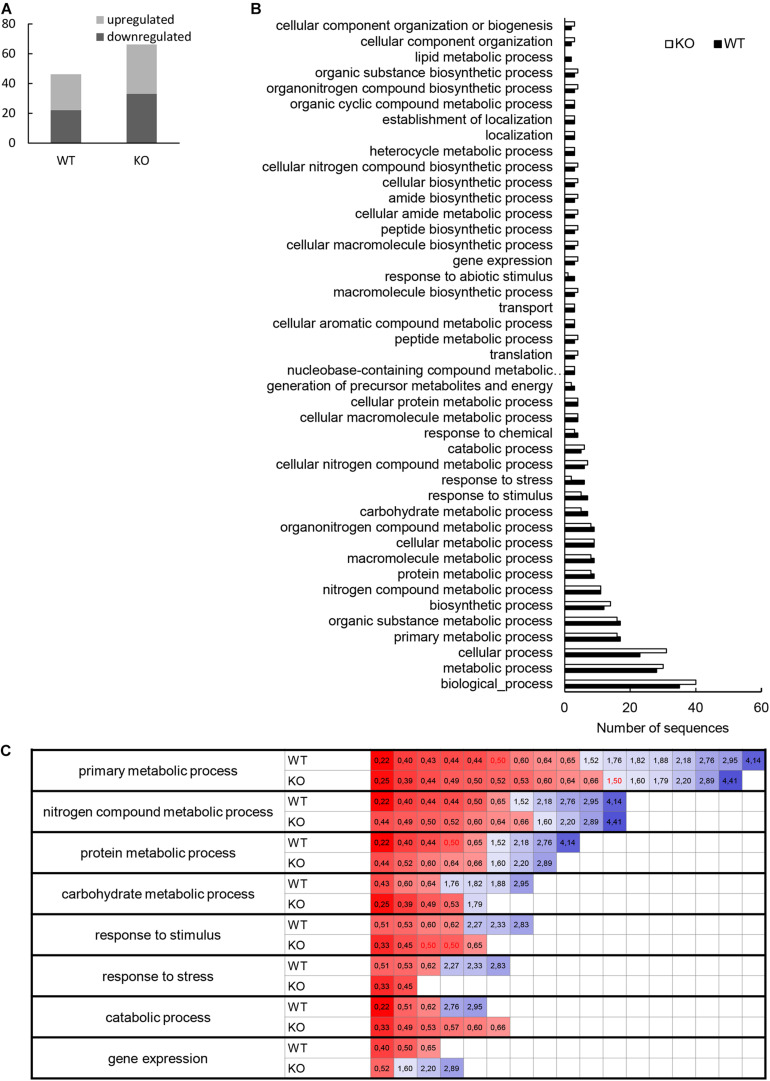
Evaluation of differentially abundant root proteins found in wild type (WT) and *HvMPK3* KO lines (KO) after flg22 treatment. **(A)** Graph showing numbers of down- and up-regulated proteins in the both lines as compared to control. **(B)** Graph showing a comparison of gene ontology annotation of differentially regulated proteins found in the analyzed lines. **(C)** Heat map showing the fold change of proteins annotated to the most important gene ontology annotations.

The comparative shot-gun proteomic analysis of flg22-treated plants resulted in identification of 53 and 58 proteins with significantly changed abundance in WT and *HvMPK3* KO plants, respectively (Figure 6A and Supplementary Data Sheets 4A, 5A). *HvMPK3* knock-out caused a remodeling of barley proteome in response to flg22, as suggested by the functional classification of the differentially abundant proteins in the wild type and *HvMPK3* KO plants (Figures 6B,C and Supplementary Data Sheets 4B–E, 5B–E).

We again compared the proteomes of wild type and KO mutants with the help of GO annotation. It clearly showed that flg22-affected proteome of the mutants contained significantly less proteins classified into annotations named response to stress and response to abiotic stimulus (Figure 6B and Supplementary Data Sheet 5B). We looked more closely at the most important metabolic processes as well as stress related proteins by taking into account the fold change of individual proteins (Figure 6C). We have found that metabolic processes (primary metabolic processes, nitrogen compound metabolic processes, protein metabolic processes and carbohydrate metabolic processes) did not change in the mutants as compared to the wild type, while they differed in proteins involved in response to stress, catabolic processes and gene expression. Unlike in the wild type, proteins involved in plant stress response and catabolic processes were downregulated in the mutants only, while proteins involved in gene expression were predominantly upregulated in the mutants, but not in the wild type (Figure 6C).

We also focused on differences in the presence of protein families within the differential proteomes (Supplementary Figure 5). We have found that ATPases with nucleotide binding domain, glutathione S-transferases and ADF-H/Gelsolin-like domain superfamily proteins were only present in the mutant differential proteomes, while the P-loop containing nucleoside triphosphate hydrolase family and proteins of thaumatin family were preferentially affected in the wild type differential proteome (Supplementary Figure 5).

**FIGURE 7:**
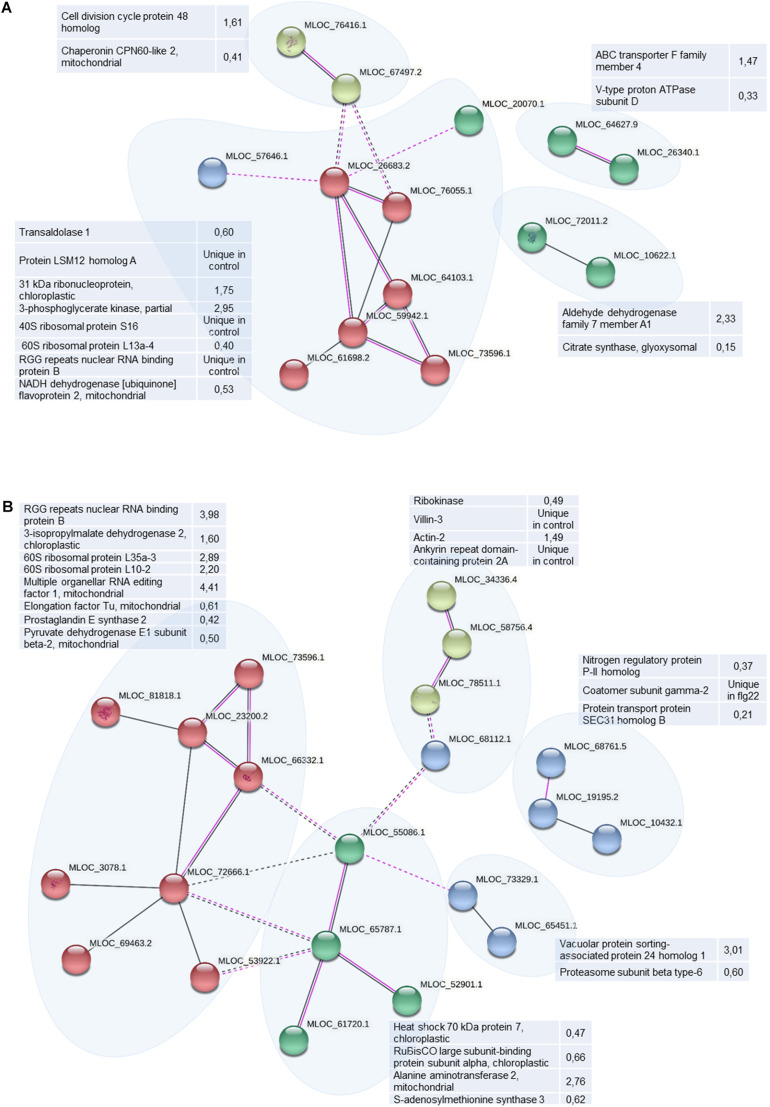
Schematic presentation of protein interaction networks in differential proteomes of flg22-treated wild type [WT; **(A)**] and *HvMPK3* KO lines **(B)** as generated by STRING web-based application.

String analysis allows to link identified proteins into functionally interconnected clusters helping to better understand the differences between large datasets. Taking into account co-expresion and experimentally found interactions among differentially abundant proteins (including their orthologues), we have found 4 protein clusters in differential proteome of WT (Figure 7A). The biggest cluster encompassed proteins involved in translation connected mostly by protein-protein interactions. The functions of 3 small clusters is unknown. Differentially abundant proteins in *HvMPK3* KO line form 5 clusters. The biggest one contains translational proteins (Figure 7B). The smaller one links cytoskeletal proteins with a metabolic ribokinase and ankyrin repeat domain-containing protein 2a, a chaperone of membrane bound proteins. This cluster shows possible functional connections to another cluster gathering chaperones and amino acid metabolic enzymes (Figure 7B). Other smaller clusters contain proteins involved in membrane transport. This analysis indicated that HvMPK3 deficiency affected protein interaction clusters involved in cytoskeleton regulation and membrane transport.

Further, we searched for predicted MAPK-specific phosphorylation sites in the amino acid sequences of differentially abundant proteins found after flg22 treatment. We have found that 54 and 49% of the differentially abundant proteins detected in WT and *HvMPK3* KO plants contain multiple (more than three) MAPK-specific phosphorylation sites, respectively (Supplementary Data Sheets 6A,B). Universal stress protein PHOS34, a protein downregulated in WT in response to flg22 was previously found by proteomic studies as MPK3/MPK6 target (Hoehenwarter et al., 2013). Furthermore, eukaryotic translation initiation factor 4B1, allene oxide cyclase, BTR1 (Binding to TOMV RNA 1) and 3-isopropylmalate dehydratase large subunit, are proteins possessing more than five MAPK-specific phosphorylation sites (Supplementary Data Sheet 6A). Among *HvMPK3* KO-specific proteins predicted to be phosphorylated by MAPKs, protein transport protein SEC31 homolog B (target of MPK3, MPK4 and MPK6) and villin-3 (phosphorylated by MPK3 and MPK6) were found as MAPK substrates experimentally (Rayapuram et al., 2018). Some other proteins, such as coatomer subunit gamma-2, nucleolin 2, heat shock 70 kda protein, cysteine synthase, dehydrin DHN5, thiol protease aleurain, histone H1, and probable inactive purple acid phosphatase 1 possess more than five MAPK-specific phosphorylation sites (Supplementary Data Sheet 6B).

More detailed elaboration of the differentially abundant proteins considering previously published information on top blast hits found for individual proteins with differential abundance, showed that together 8 of them are connected to plant defense response in flg22-treated wild type, all with significantly increased abundances (Table 2). Among them were PR protein 4, papain-like cysteine proteinase, 23 kDa jasmonate-induced protein, thaumatin-like protein TLP7, basic pathogenesis-related protein PR5, all recognized as canonical barley defense-related proteins. In addition, 12-oxophytodienoate reductase 7, involved in jasmonic acid biosynthesis was also upregulated. In opposite, two secretory peroxidases with possible defense-related functions showed reduced abundances in wild type response to flg22.

**TABLE 2:** Differentially abundant defense related proteins found in flg22-treated barley wild type roots.

Accession	MW (kDa)	calc. pI	Description	Ratio (*HvMPK3* KO vs. wild type)	*P*-value
326506982	91.7	6.48	Beta-galactosidase 6 (*Oryza sativa* subsp. *japonica*)”	0.43	0.046
326491407	36	7.8	PER72 Peroxidase 72 (*Arabidopsis thaliana*)	0.51	0.009
326501536	24	8.35	PER15 Peroxidase 15 (*Arabidopsis thaliana*)	0.62	0.036
194352756	51.1	5.36	papain-like cysteine proteinase (*Hordeum vulgare* subsp. *vulgare*)	1.52	0.013
326527541	90.1	5.29	CDC48 Cell division cycle protein 48 homolog (*Glycine max*)	1.61	0.021
326491679	83.5	5.96	BXL6 Probable beta-D-xylosidase 6 (*Arabidopsis thaliana*)	1.76	0.030
326508800	32	5.48	Chitinase 2 (*Tulipa saxatilis* subsp. *bakeri*)	1.88	0.024
326527975	23.1	8.65	23 kDa jasmonate-induced protein (*Hordeum vulgare*)	2.33	0.018
14164981	23.6	7.34	thaumatin-like protein TLP7 (*Hordeum vulgare*)	2.43	0.019
2344818	25.2	6.83	basic pathogenesis-related protein PR5 (*Hordeum vulgare* subsp. *vulgare*)	2.83	0.011
15797690	25.9	8.82	AOC Allene oxide cyclase, chloroplastic (*Oryza sativa* subsp. *japonica*)	Unique in control	NA
326528939	38.1	6.39	BTR1 Protein BTR1 (*Arabidopsis thaliana*)	1.76	0.046

Next, we encountered an increased abundance of lipoxygenase 2 and glucan endo-1,3-beta-glucosidase, a cell wall modifying enzyme likely involved in defense response in *HvMPK3* KO plants. In contrast, pathogenesis-related protein PRMS was downregulated (Table 3).

**TABLE 3:** Differentially abundant defense related proteins found in flg22-treated barley *HvMPK3* KO roots.

Accession	MW [kDa]	calc. pI	Description	Ratio (*HvMPK3* KO vs. wild type)	*P*-value
326488925	33	7.93	Glucan endo-1,3-beta-glucosidase GI (Hordeum vulgare)	1.79	0.045
2429087	96.7	6.73	lipoxygenase 2 [Hordeum vulgare subsp. vulgare]	Unique in control	NA
326529301	18.7	9.11	PRMS Pathogenesis-related protein PRMS (Zea mays)	0.21	0.020
326518626	37.1	8.02	PER1 Peroxidase 1 (Zea mays)	0.33	0.030

**FIGURE 8:**
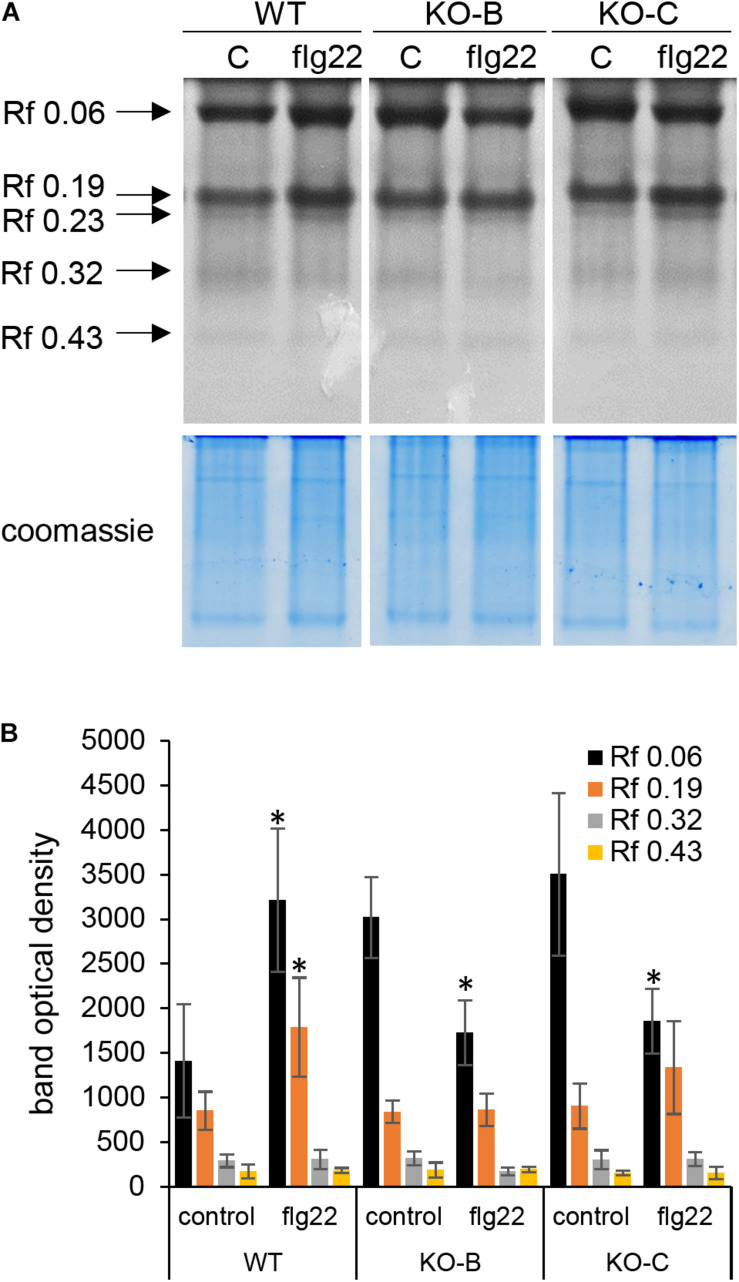
Examination of chitinase activity in the roots of wild type (WT) and *HvMPK3* KO lines (KO-B and KO-C) in response to flg22. **(A)** Chitinase activity on native PAGE gels. Arrows indicate chitinase isozymes with designated relative mobility (Rf). Right panel: visualization of proteins on gel in A by Coomassie staining. **(B)** Quantification of band intensities in **(A)**. Asterisks indicate statistically significant difference between flg22-treated and control samples. Uncropped, full original image of the gel is documented in Supplementary Figure 9B.

We again used the opportunity to examine the chitinase activity in wild type and mutant lines after flg22 treatment. This analysis showed differential response of individual chitinase isoforms to flg22, revealing upregulation of isoform with Rf 0.06 in the wild type, but significant downregulation in both mutant lines. Activity of isoform with Rf 0.19 was also increased in the wild type, but it did not show significant changes in the mutants (Figure 8). Our data suggest that unlike in the wild type, activities of some chitinase isoforms failed to respond to flg22 in the *HvMPK3* KO mutants.

These results clearly show that flg22 elicited a defense response in the wild type. *HvMPK3* deficiency leads to the alleviation of this elicitation, implicating that this MAPK is essential for induction of primary immune response in barley.

### Root Hair Phenotype and Its Response to flg22 in the *HvMPK3* KO Mutants

Immune and developmental signaling pathways are tightly interconnected and vital trade off mechanism occur in plants balancing these two programs (Naseem et al., 2015; Scheres and van der Putten, 2017). The immense changes in abundances of proteins involved in polarized growth prompted us to focus on root hair formation in *HvMPK3* KO lines. Root hairs facilitate efficient water and nutrient uptake from the soil and belong to important agronomic traits in crops.

**FIGURE 9:**
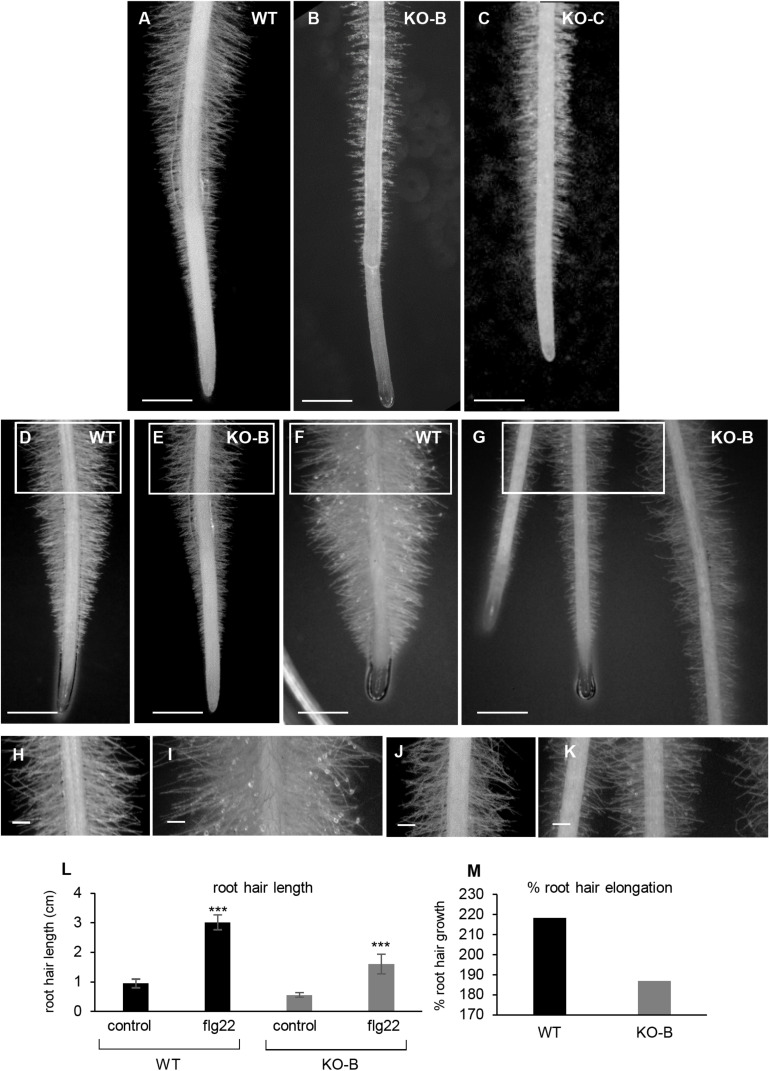
Influence of *HvMPK3* knock-out and flg22 treatment on the phenotype of root hairs. **(A–C)** Comparison views of root apex between wild type [WT; **(A)**] and *HvMPK3* KO-B **(B)** and KO-C **(C)** lines showing decreased root hair elongation. **(D–G)** Overview of control WT **(D,F)** and *HvMPK3* KO-B **(E,G)** roots 2 days after the transfer to either control media **(D,E)** or flg22-containing media **(F,G)**. Note, that WTs develop dense and highly elongated root hairs compared to *HvMPK3* KO roots. **(H–K)** Higher magnification of the boxed areas of **(D–G)** showing in detail terminally grown root hairs of control WT and *HvMPK3* KO **(H,J)** and after flg22 treatment **(I,K)**. **(L,M)** Quantitative assessment of root hair length **(L)** and percentage of increased root hair elongation **(M)** comparing control and flg22-treated WT and *HvMPK3* KO seedlings. Scale bars: 2 mm **(A–G)**, 500 μm **(H–K)**; ***p < 0.001, Student’s *t*-test.

Qualitative and quantitative comparison of root hair phenotypes showed pronounced difference between wild type and *HvMPK3* KO seedlings. Wild type roots exhibit the typical fir-tree appearance of root hairs (Figure 9A) with emerging root hairs at the root differentiation zone and progressively elongating at increasing distances from the root apex. The length of terminally elongated root hairs in wild type roots was highly variable and averaged at 0.946 ± 0.148 mm (*N* = 431 root hairs). By contrast, terminally elongated root hairs of the *HvMPK3* KO mutants (Figures 9B,C) were 0.558 ± 0.075 mm (*N* = 404 root hairs). Transfer of wild type seedlings to solid medium supplemented with 1 μM flg22 resulted in over-elongation of root hairs (Figures 9D–G). By contrast to what was reported for *Arabidopsis* (Pečenková et al., 2017; Okada et al., 2021), where flg22 treatment does not significantly affect root hair formation, it seems that exogenous application of flg22 strongly stimulated root hair growth in both barley genotypes (Figures 9D–M). The length of WT root hairs following exposure to 1 μM flg22 for 2 days was 3.01 ± 0.25 mm (mean ± SD, *N* = 421 root hairs; Figure 9L). Root hairs of *HvMPK3* KO seedlings were also responsive to exogenous flg22 albeit to a lesser extent. Thus, *HvMPK3* KO root hairs were 1.6 ± 0.33 mm long (mean ± SD, *N* = 410 root hairs; Figure 9L). By average, WT root hairs elongated by 318.18% while those of *HvMPK3* KO elongated by 286.74% (Figure 9M) upon flg22 treatment, as compared to control condition.

## Discussion

Mitogen activated protein kinase signaling lies at the core of extracellular stimuli perception and in plants it has diversified extensively to incorporate the largest families of MAPKKKs, MAPKKs, and MAPKs (Komis et al., 2018). From prevalent studies in dicots exemplified in the model plant *A. thaliana*, MAPK cascades have been found to positively or negatively regulate innate immune responses. In *Arabidopsis*, PAMPs activate simultaneously two distinct MAPK pathways, namely MKKK3/5–MKK4/5–MPK3/6 and MEKK1–MKK1/2–MPK4 (Asai et al., 2002; Suarez-Rodriguez et al., 2007; Kong et al., 2012). Upon *Arabidopsis* infection with *Botrytis cinerea*, AtMPK3/MPK6 pair phosphorylates the AtWRKY33 transcription factor in order to trigger expression of camalexin biosynthetic genes (Mao et al., 2011; Zhou et al., 2020). Both kinases also regulate ethylene production at the same conditions via phosphorylation of 1-amino-cyclopropane-1-carboxylic acid synthase (Han et al., 2010), and positively regulate expression of many defense-related genes (including basic endochitinase) by phosphorylation of ERF6 transcription factor upon *B. cinerea* infection (Meng et al., 2013). Thus, *Arabidopsis* MPK3 and MPK6, as well as their homologues in other species confer resistance to pathogens in dicots and monocots (El Sarraf et al., 2019; Kim et al., 2019; Liu et al., 2019; Wang et al., 2019).

### The Impact of HvMPK3 on flg22-Induced Immune Responses

Genetic modification of MAPKs affects either plant basal or induced immune defense (Hoehenwarter et al., 2013; Rayapuram et al., 2018). This capability differs depending on particular MAPK species. For example, *Arabidopsis* MPK6 controls the elicitor-induced resistance while MPK3 has impact on basal resistance to *B. cinerea* (Ren et al., 2008; Galletti et al., 2011). Comprehensive transcriptomic study on *Arabidopsis* MAPK mutants showed that the reduction in the flg22 response could not be explained by the basal transcriptome changes observed in these mutants in the absence of stress (Frei dit Frey et al., 2014). According to our analyses, HvMPK3 determines both the basal and induced immune response in barley, mainly by regulation of abundance of PR proteins (including chitinases) and glutathione S-transferases. Thus, HvMPK3 shows impact on flg22-induced defense activation, unlike *Arabidopsis* MPK3.

One of the most striking findings in the basal proteome of *HvMPK3* KO mutants, is the downregulation of secretory peroxidases in roots and shoots of *HvMPK3* KO seedlings. The impact of MAPKs on the expression and abundance of secretory peroxidases, was already reported. *Arabidopsis mpk6* mutant shows upregulation of PRX34 and increased apoplastic H_2_O_2_ production (Han et al., 2015). Changes in abundance and activities of peroxidases were found in a shot-gun proteomic study of *Arabidopsis mpk4* and *mpk6* mutants as well (Takáč et al., 2016). We show that in addition to MPK4 and MPK6 of *Arabidopsis*, barley HvMPK3 has remarkable impact on basal expression of secretory peroxidases. Only very limited information is available about the regulation of secretory peroxidases. Their transcriptional control is mediated by AT-hook protein OsATH1 in rice (Liu et al., 2021) or by AGAMOUS-LIKE15 (AGL15) in *Arabidopsis* (Cosio et al., 2017). The mechanism of MAPK-dependent regulation of peroxidases is not resolved yet. However, their direct phosphorylation cannot be excluded, as exemplified by the presence of six MAPK-specific phosphorylation sites in the amino acid sequence of PEROXIDASE 1. In addition, PEROXIDASE 52 was phosphorylated on MAPK-specific SP motif in untreated WT roots.

As found by proteomic studies, plants respond to elicitor treatment by upregulation of PR proteins, glutathione S-transferases, enzymes of phytohormone (jasmonic acid, ethylene, auxin) biosynthesis (Chivasa, 2006; Casasoli et al., 2008), calcium signaling (Lippert et al., 2009; Zulak et al., 2009), and by downregulation of chaperones (Chivasa, 2006). In agreement, WT barley roots in our experimental system responded to flg22 treatment by increased abundance of PR proteins and glutathione S-transferases. Knockout of *HvMPK3* compromised the synthesis of defense related proteins, and caused downregulation of chloroplastic and mitochondrial HSP70 proteins. This might be linked to known MPK3/6-dependent phosphorylation of heat stress transcription factor A1 (HSFA1) and HSFA4 in *Arabidopsis* (Evrard et al., 2013; Pérez-Salamó et al., 2014), two master regulators of plant heat stress response controlling the expression of multiple heat shock proteins. Nevertheless, the MAPK-mediated regulation of HSP70 abundance during immune response was not reported so far. HSP70 promotes plant defense and its activity and localization is altered by binding to HOP1, a *Pseudomonas syringae* effector in temperature dependent manner (Jelenska et al., 2010). Our data indicate a new role of HvMPK3 in flg22-induced abundance of HSP70. One of the possible mechanisms of HSP70 regulation includes its phosphorylation as shown by the prediction of MAPK-specific phosphorylation sites.

Plant chitinases are cell wall localized chitin-degrading enzymes induced during plant defense response to pathogens, but also by diverse environmental conditions including heavy metals (Békésiová et al., 2008), cold stress (Yeh et al., 2000; Nakamura et al., 2008) or salt stress (Takenaka et al., 2009). The expression of chitinases is induced by elicitation with chitin (Brunner et al., 1998), but also by other elicitors, such as flg22 (Ohnuma et al., 2011; Trdá et al., 2014; Meng et al., 2019). Here, we show, that unlike to the wild type, chitinases were not differentially abundant in roots of *HvMPK3* KO lines in response to flg22. Moreover, this was also supported at the level of chitinase activity. These results suggest that chitinases, likely in isoform specific manner, are positively regulated by HvMPK3 upon flg22 elicitation.

Actin cytoskeleton plays a well-established role during plant immune responses. Receptor kinase-dependent increase in actin filament abundance during immune response (Henty-Ridilla et al., 2013) is dependent on inhibition of actin severing by actin depolymerizing factor 4 (ADF4) (Henty-Ridilla et al., 2014) and by actin capping protein (Li et al., 2015) during elicitation. Our results show that *HvMPK3* deficiency is linked with enhanced abundance of actin depolymerization factor-like protein during immune response. Moreover, attenuated responsiveness of *HvMPK3* KO plants to flg22 is accompanied also by decreased abundance of actin bundling protein VLN3. This shows that absence of HvMPK3 may lead to defects in proper actin organization during immune response. VLN3, unlike to ADF-like protein, contains multiple phosphorylation sites in its amino acid sequence and was detected as a phosphorylation target of *Arabidopsis* MPK3 and MPK4 (Rayapuram et al., 2018). This implies that VLN3 is directly regulated by MAPKs, and ADF-like protein exerts indirect regulation by MAPKs.

### Actin Binding Proteins, Membrane Transport Regulatory Proteins and Also Peroxidases Likely Contribute to the Root Hair Defects in *HvMPK3* KO Plants

Actin cytoskeleton is essential for multiple developmental processes including polarized tip growth of root hairs (Šamaj et al., 2006; Bascom et al., 2018). Plant growth and defense signaling are closely connected (Naseem et al., 2015). In this regard, MPK3 is a possible candidate linking these signal-dependent events.

A brief outlook of *HvMPK3* KO phenotype shows that likely MPK3 is involved in root hair development, but most importantly, exhibits a degree of resilience, but not complete resistance to the exogenous application of flg22. It might be hypothesized, that this partial resistance is due to the presence of HvMPK6 in *HvMPK3* KO plants, as these kinases often work in the same pathway (Asai et al., 2002).

Root hair production seen in WT was partially abrogated in *HvMPK3* KO lines, suggesting that sensitivity to flg22 elicitation is mediated by MPK3 as is the case in *Arabidopsis*. Earlier studies have demonstrated that at least in *Arabidopsis*, MPK3 may have variable roles in innate immunity and elicitor-triggered resistance, having either positive (Galletti et al., 2011) or negative effects (Frei dit Frey et al., 2014). The exogenous application of different bacterial elicitors was previously shown to affect root growth in *Arabidopsis*, by inhibiting elongation at a variable extent (Poncini et al., 2017). Albeit less potent than AtPep1, flg22 may promote root growth inhibition at significant rates when applied at concentrations similar to those used herein (100 nM–1 μM) for prolonged periods of time (Gómez-Gómez et al., 1999; Poncini et al., 2017; Garrido-Oter et al., 2018).

Previous shot-gun proteomic analysis of *mpk4* and *mpk6* mutants of *Arabidopsis* showed deregulation of defense-regulated proteins and altered abundance of proteins involved in diverse developmental processes, correlating with mutant phenotypes (Takáč et al., 2016). The root hair branching and ectopic root hair formation in *mpk4* mutant of *Arabidopsis* (Beck et al., 2010) is accompanied by alteration in membrane transport regulatory proteins as well as actin binding proteins such as profilins, dehydrin ERD10 or annexin 1 (Takáč et al., 2016). Our proteomic study reveals that root proteomes of barley *HvMPK3* KO lines possess deregulated adaptor proteins involved in recruitment of clathrin to membranes (beta-adaptin-like protein C, containing clathrin adaptor, alpha/beta/gamma-adaptin appendage domain), transport from ER to Golgi apparatus (protein transport protein SEC31 homolog B; Takagi et al., 2013), and alpha-soluble NSF attachment protein (Bayless et al., 2016). Like other membrane transport regulatory proteins, also those controlling ER to Golgi transport are inevitable for proper root hair formation and elongation (Martinière and Moreau, 2020). We also observed a downregulation of protein heterologous to *Arabidopsis* VILLIN 2. Villins are actin bundling proteins involved in root elongation and root hair development (Zhang et al., 2011; van der Honing et al., 2012). Plants of *villin2villin3* double mutant in *Arabidopsis* show decreased number of thick actin bundles, both in tip-growing (van der Honing et al., 2012) and diffusely growing cells (Bao et al., 2012), supporting an important role of villins in regulating cell expansion through bundling of actin filaments. In tip-growing cells, such as root hairs and pollen tubes, different roles of villins in the regulation of actin dynamics have been suggested. This is based on the differential actin organization in apical, subapical and shank regions of the tip-growing cell (Staiger et al., 2010; Huang et al., 2015). The importance of villins have been extrapolated by observations in pollen tubes of *Arabidopsis villin2villin5* double mutant exhibiting much slower tip growth compared to wild type, or single villin mutants. Double mutants showed disruption of actin filaments and this disorganization caused that vesicles labeled with the RabA4b marker, were not transported to the apical and subapical regions of pollen tubes correctly (Qu et al., 2013). Recently, it was documented that the important role of VILLIN1 in the root hair growth of *Arabidopsis*, is mediated by the transcription factor GLABRA2, and this transcriptional regulation can be involved also in modulation of actin dynamics in root hairs under osmotic stress (Wang et al., 2020). Interestingly, VILLIN 3 was downregulated in *HvMPK3* KO lines also upon flg22 treatment. Importantly, as noted above, villins belong to identified AtMPK3 phosphorylation targets (Rayapuram et al., 2018), which is further strengthening the hypothesis about the villin-mediated defects of root hair formation in *HvMPK3* KO plants.

As noted above, *HvMPK3* KO roots showed reduced abundance of four secretory peroxidase isoforms. These enzymes with H_2_O_2_ decomposing but also H_2_O_2_ forming activity are induced in plants during pathogen response in order to crosslink cell wall components leading to cell wall reinforcement (Almagro et al., 2009). They were also linked to H_2_O_2_-mediated root elongation by elevation of H_2_O_2_ levels under the control of MPK6 in *Arabidopsis* (Han et al., 2015). Peroxidase-mediated ROS production also correlates with root hair initiation in barley (Kwasniewski et al., 2013). These data indicate that peroxidases regulated by HvMPK3 may contribute to root hair growth in barley. The unique dynamics of peroxidases might contribute to different response of barley WT showing induction of root hair formation by flg22, unlike to *Arabidopsis* (Okada et al., 2021).

Considering MAPK developmental roles in root hair formation, these are known solely for *Arabidopsis* MPK4 (Beck et al., 2010) and *Medicago sativa* SIMK, which is an orthologue of *Arabidopsis* MPK6 (Šamaj et al., 2002; Hrbáčková et al., 2020, in press) so far. This study is the first one reporting about the involvement of MPK3 in root hair growth.

## Data Availability Statement

The original contributions presented in the study are included in the article/Supplementary Material, further inquiries can be directed to the corresponding author. The mass spectrometry proteomics data have been deposited to the ProteomeXchange Consortium via the PRIDE (Perez-Riverol et al., 2019) partner repository with the dataset identifier PXD022913.

## Author Contributions

PKř, PKa, LO, and TTi generated and selected transgenic barley lines used herein. TTa and TP prepared and conducted proteomics analysis. MA created a script for protein quantitative analysis. TTa, MO, JB, and GK conducted phenotypic documentation and analysis. TTa and PV conducted biochemical analyses. TTa, PKř, GK, and JŠ drafted the manuscript with input from all co-authors. JŠ conceived and supervised the project, provided infrastructure and secured funding. All authors contributed to the article and approved the submitted version.

## Conflict of Interest

The authors declare that the research was conducted in the absence of any commercial or financial relationships that could be construed as a potential conflict of interest.

## Footnotes

1 http://plants.ensembl.org/index.html

2 https://www.ncbi.nlm.nih.gov/

3 https://blast.ncbi.nlm.nih.gov/Blast.cgi

4 https://jorgensen.biology.utah.edu/wayned/ape/

5 http://wormweb.org/exonintron

6 https://tale-nt.cac.cornell.edu/

## Supplementary Material

Supplementary Figure 1 Transcript comparison of the HORVU4Hr1G057200 *HvMPK3* gene.

Supplementary Figure 2 Alignment of the barley HvMPK3 and *Arabidopsis* AtMPK3 amino acid sequences.

Supplementary Figure 3 KEGG pathway analysis of differentially abundant proteins found between roots of *HvMPK3* KO lines and wild types.

Supplementary Figure 4 Gene ontology annotation analysis of differentially abundant proteins found between above ground parts of *HvMPK3* KO lines and wild types.

Supplementary Figure 5 Evaluation of protein families in the differential proteomes of wild type and *HvMPK3* KO roots.

Supplementary Figures 6–9 Full original images of immunoblotting membranes and gels presented in Figures 2, 4, 5, 8.

Supplementary Table 1 Overview of the oligonucleotides used in this study.

Supplementary Data Sheet 1 Differentially regulated proteins found between roots of wild type and *HvMPK3* KO lines and their bioinformatic evaluation.

Supplementary Data Sheet 2 Differentially regulated proteins found between above ground parts of wild type and *HvMPK3* KO lines and their bioinformatic evaluation.

Supplementary Data Sheet 3 Phosphopeptides detected in the proteomes of untreated wild type and *HvMPK3* KO line roots and leaves.

Supplementary Data Sheet 4 Differentially regulated proteins found between controls and flg22-treated roots of barley wild type lines and their bioinformatic evaluation.

Supplementary Data Sheet 5 Differentially regulated proteins found between controls and flg22-treated roots of barley *HvMPK3* KO lines and their bioinformatic evaluation.

Supplementary Data Sheet 6 Prediction of MAPK-specific phosphorylation sites and docking motifs in amino acid sequences of differentially abundant proteins found in flg22-treated wild type and *HvMPK3* KO mutant roots.
