# Supplementary material for: TALEN-Based HvMPK3 Knock-Out Attenuates Proteome and Root Hair Phenotypic Responses to flg22 in Barley
Source: Front Plant Sci. 2021 Apr 29;12:666229. doi: 10.3389/fpls.2021.666229 (PMC8117018; doi:10.3389/fpls.2021.666229)
Supplement: Supplementary Figure 1 — Transcript comparison of the HORVU4Hr1G057200 HvMPK3 gene. [file Presentation_1.pdf]

Supplementary Material

TALEN-based *HvMPK3* knock-out attenuates proteome and root hair phenotypic responses to flg22 in barley

Tomáš Takáč[1#], Pavel Křenek[1#], George Komis[1], Pavol Vadovič[1], Ludmila Ohnoutková[2], Tibor Pechan[3], Petr Kašpárek[4], Tereza Tichá[1], Miroslav Ovečka[1], Jasim Basheer[1], Mark Arick II[3], Jozef Šamaj[1]*

[1]Department of Cell Biology, Centre of the Region Haná for Biotechnological and Agricultural Research, Faculty of Science, Palacký University Olomouc, Olomouc, Czech Republic

[2]Laboratory of Growth Regulators, Palacký University & Institute of Experimental Botany, Czech Academy of Sciences, Olomouc, Czech Republic

[3]Institute for Genomics, Biocomputing and Biotechnology, Mississippi Agricultural and Forestry Experiment Station, Mississippi State University, Starkville, MS, United States

[4]Laboratory of Transgenic Models of Diseases, Institute of Molecular Genetics of the CAS, v.v.i, 252 50 Vestec, Czech Republic

[#] these two authors contributed equally to this work

Correspondence: jozef.samaj@upol.cz

Table S1. Overview of the oligonucleotides used in this study

Method	Gene/Linker	Oligo name	5'-3' sequence
Linker preparation	Acc65I-NheI	Link1_F	GTACCagtcttgtgagtG
		Link1_R	CTAGCactcacaagactG
	NotI-SbfI	Link2_F	GGCCGCacttgcaagaCCTGCA
		Link2_R	GGtcttgcaagtGC
T-DNA genotyping	*hpt*	hptF	ACTCACCGCGACGTCTGT
		hptR	GCGCGTCTGCTGCTCCAT
Mutation genotyping	*HvMPK3*	K3F1	CGGTTTGGTTCTTGGCTGTT
		K3R1	ACCACAGAGCACCGACAGAT
RT-qPCR	*HvMPK3*	qK3F1	GTAAGATCGAAGAACGGGGTTA
		qK3R1	AGGTCTGAAGCAGCAGCAA
	HvMPK14	qK14F1	CACAAAAGCCACGCAGAGA
		qK14R1	CCGAACCAACCACTTTACCA

Script used for the relative quantification of proteins.

```
#!/usr/bin/env perl

use strict;
use warnings;
use autodie;

use Data::Dumper;

use Tk;
use Tk::DropSite;
use Spreadsheet::ParseExcel;
use Spreadsheet::ParseXLSX;
use Statistics::TTest;

use List::Util qw/sum0 sum reduce/;
use File::Basename qw/ fileparse /;
use File::Spec;


my $elements = {};
my $savefile = "";
my $normalize = 1;
my $filter = 0;
my $filter_num = 5;

my $mw = MainWindow->new(title => "Intensity Ratio");

$mw->Label(-text => 'Drag files onto the list boxes below or open them individually:')->pack;

$mw->Button(
    -text    => 'Run',
    -command => ¥&run_button,
    )->pack(-side=>'bottom', -anchor=>'e');


my $filter_frame = $mw->Frame()->pack(-fill=>'x', -side=>"bottom");
$filter_frame->Checkbutton(
    -text    => 'Normalize Intensity',
    -variable => ¥$normalize,
    )->pack(-side=>'right');
$filter_frame->Checkbutton(
    -text    => 'Filter Most Intense Spetra',
    -variable => ¥$filter,
```

```
    )->pack(-side=>'left');
$filter_frame->Entry(-textvariable => ¥$filter_num)->pack(-anchor=>'w');




my $out_frame = $mw->Frame()->pack(-fill=>'x', -side=>"bottom");
$out_frame->Label(-text => 'Output File:')->pack(-side => 'left');
$out_frame->Button(-text=>'Save', -command=>¥&save_button)->pack(-anchor=>'w', -side=>'right');
$out_frame->Entry(-textvariable => ¥$savefile)->pack(-fill=> 'x', -side => 'top', -expand=>1);

my $df = $mw->Frame()->pack(-expand=>1, -fill => 'both');
foreach (qw/A B/){
    my $frame = $df->Frame(
        -borderwidth => 1, #A frame title
        -relief => 'ridge',
        )->grid(-sticky => "nsew");
    my $f = $frame->Frame()->pack(-fill=>'x');

    $f->Label(-text => 'Treatment Name:')->pack(-side => 'left');
    $f->Button(-text=>'Open', -command => [¥&open_button, $_])->pack(-anchor=>'w', -side=>'right');
    $elements->{label}->{$_} =$f->Entry()->pack(-fill=> 'x', -side => 'top', -expand=>1);

    $elements->{files}->{$_} = $frame->Listbox()->pack(-expand=>1,  -fill => 'both');
}

$df->gridRowconfigure(0, -weight=>1, -uniform=>"group1");
$df->gridRowconfigure(1, -weight=>1, -uniform=>"group1");
$df->gridColumnconfigure(0, -weight=>1);

MainLoop;


sub open_button {
    my $section = shift;
    my $files = $mw->getOpenFile( -filetypes => [
                                       ['XLS[X] - Excel Files', ['*.XLS', '*.xls','*.XLSX',
'*.xlsx']],
                                       ['All Files - *', '*']
                                       ],
                                  -title => 'Select a file',
                                  -multiple=>1
        );

    $elements->{files}->{$section}->insert('end', @$files);
}
sub save_button {
    $savefile = $mw->getSaveFile( -filetypes => [
```

```
                                    ['XLS - Excel Files', ['*.XLS', '*.xls']],
                                    ['All Files - *', '*']
                               ],

                                 -title => 'Select a file',
                                 -multiple=>1
        );

}
sub run_button {
    my $files = {};
    my $labels = {};

    while (my ($k, $v) =  each %{$elements->{files}}){
        $files->{$k} = [$v->get(0, 'end')];

        die "$k has empty file list" unless @{$files->{$k}};

        foreach(@{$files->{$k}}){
            die "$_ doesn't exist" unless ( -e $_ );
        }
    }

    while (my ($k, $v) =  each %{$elements->{label}}){
        $labels->{$k} = $v->get() || $k;
    }

    my $data = { };
    my $desc = {};
    my $desc_header = ['MW [kDa]', 'calc. pI', 'Description'];


    foreach my $set (qw/ A B /){
        foreach my $file ( @{$files->{$set}}){
            my $parser   = Spreadsheet::ParseExcel->new();
            my $workbook = $parser->Parse($file);

            if ( !defined $workbook ) {
                $parser = Spreadsheet::ParseXLSX->new();
                $workbook = $parser->parse($file);
            }

            if ( !defined $workbook ) {
                die $parser->error(), ".¥n";
            }

            my $worksheet = $workbook->worksheet(0);
```

```
        my ( $row_min, $row_max ) = $worksheet->row_range();
        my ( $col_min, $col_max ) = $worksheet->col_range();

        my $proheader = { map {(($worksheet->get_cell($row_min, $_))?
                                $worksheet->get_cell($row_min, $_)->value:$_), $_}
                          ($col_min .. $col_max)};
        $row_min++;

        my $pepheader;
        my $accession;
        for(my $row = $row_min; $row <= $row_max; $row++) {
            if($worksheet->get_cell($row, 0) && $worksheet->get_cell($row, 0)->value){
                $accession = $worksheet->get_cell($row, $proheader->{'Accession'})->value;

                unless($desc->{$accession}){
                    $desc->{$accession} = {};
                    @{$desc->{$accession}}{@$desc_header} = map {(($worksheet->get_cell($row,
$_))?
                                                                   $worksheet->get_cell($row,
$_)->value:undef)}
                                                                  @$proheader{('MW [kDa]', 'calc.
pI', 'Description')};
                }

                $row++;
                $pepheader = [map {(($worksheet->get_cell($row, $_))?
                                    $worksheet->get_cell($row, $_)->value:$_)}
                                   ($col_min .. $col_max)];

                next;
            };

            next unless $pepheader;
            my $tmp = {};
            @$tmp{@$pepheader} = map {(($worksheet->get_cell($row, $_))?
                                        $worksheet->get_cell($row, $_)->value:undef)} ($col_min ..
$col_max);

            next unless $tmp->{"Intensity"};


            $data->{$accession} ||= {} ;
            $data->{$accession}->{$set} ||= {} ;
            $data->{$accession}->{$set}->{$file} ||= [];
            push(@{$data->{$accession}->{$set}->{$file}},
                    {Intensity => $tmp->{"Intensity"},
                     Sequence => ($tmp->{"Sequence"} || $tmp->{"Annotated Sequence"})});
        }
```

```
    }
}


my $factor = {};
while(my ($accession, $data) = each(%$data) ){
    foreach my $set (qw/ A B /){
        while(my ($file, $pep) = each(%{$data->{$set}})){
            $factor->{$file} += sum0(map{$_->{"Intensity"}} @$pep);
        }
    }
}
my $intensity_average = sum(values %$factor)/scalar(keys %$factor);
$factor = {map {$_ => $intensity_average / $factor->{$_} } keys %$factor};

$factor = {map {$_ => 1 } keys %$factor} unless($normalize);
print Dumper $factor;



my $output = {};
while(my ($accession, $data) = each(%$data) ){
    my $stats = {};
    foreach my $set (qw/ A B /){
        my $data = $data->{$set} || {};

        my $sums = [];

        if($filter){
            while ( my ($file, $peps) = (each  %$data)){
                my $filtered_sum = {};
                foreach my $pep (@$peps){
                    $filtered_sum->{$pep->{Sequence}} ||= 0;
                    if($filtered_sum->{$pep->{Sequence}} < $pep->{Intensity}){
                        $filtered_sum->{$pep->{Sequence}} = $pep->{Intensity};
                    }
                }

                $filtered_sum = [sort {$b <=> $a} values %$filtered_sum];
                my $sum = sum0(splice @$filtered_sum, 0, $filter_num);
                $sum *= $factor->{$file};

                push(@$sums, $sum);
            }

        }else{
            while ( my ($file, $peps) = (each  %$data)){
                my $sum = sum0(map { $_->{"Intensity"} } @$peps);
```

```
                    $sum *= $factor->{$file};

                    push(@$sums, $sum);
                }
            }


            my ($mean, $variance, $count) = (0,0,scalar @$sums);

            $mean = sum0(@$sums)/$count if $count;

            if($count > 1){
                $variance = reduce {$a + ($b-$mean)**2} (0, @$sums);
                $variance /= $count;
            }

            $stats->{$set} = {
                'count'    => $count,
                    'mean'     => $mean,
                    'variance' => $variance
            };

        }

        $data = {ratio => 0, pvalue => -1, stats=>$stats};

        if ($stats->{'A'}->{count} > 0 && $stats->{'B'}->{count} > 0){
            $data->{ratio} = ($stats->{'A'}->{mean}/$stats->{'B'}->{mean});
        }else{
            $data->{ratio} = "Unique in " . $labels->{(($stats->{'A'}->{count} > 0)?'A':'B')};
        }

        if ($stats->{'A'}->{count} >= 2 && $stats->{'B'}->{count} >= 2){
            my $ttest = new Statistics::TTest::Sufficient;
            $ttest->load_data($stats->{A}, $stats->{B});
            $data->{pvalue}= $ttest->{t_prob};
        }

        $output->{$accession} = $data;
    }


    open(my $out, '>', $savefile);

    print $out join "¥t", 'Accession', @$desc_header, qw(Ratio P-value A-mean A-variance B-mean B-
variance);
    print $out "¥n";
    foreach my $accession (sort {$output->{$a}->{pvalue} <=> $output->{$b}->{pvalue}}
```

```
keys %$output){
        my $data = $output->{$accession};
        print $out join( "¥t", $accession, @{$desc->{$accession}}{@$desc_header},
                         $data->{ratio}, (($data->{pvalue} == -1)?"NA":$data->{pvalue}),
                    #                ($adjusted->{$accession} || "NA"),
                map {@$_{qw/ mean variance /}} @{$data->{stats}}{qw/A B/}) . "¥n";
    }
    close $out;

}
```

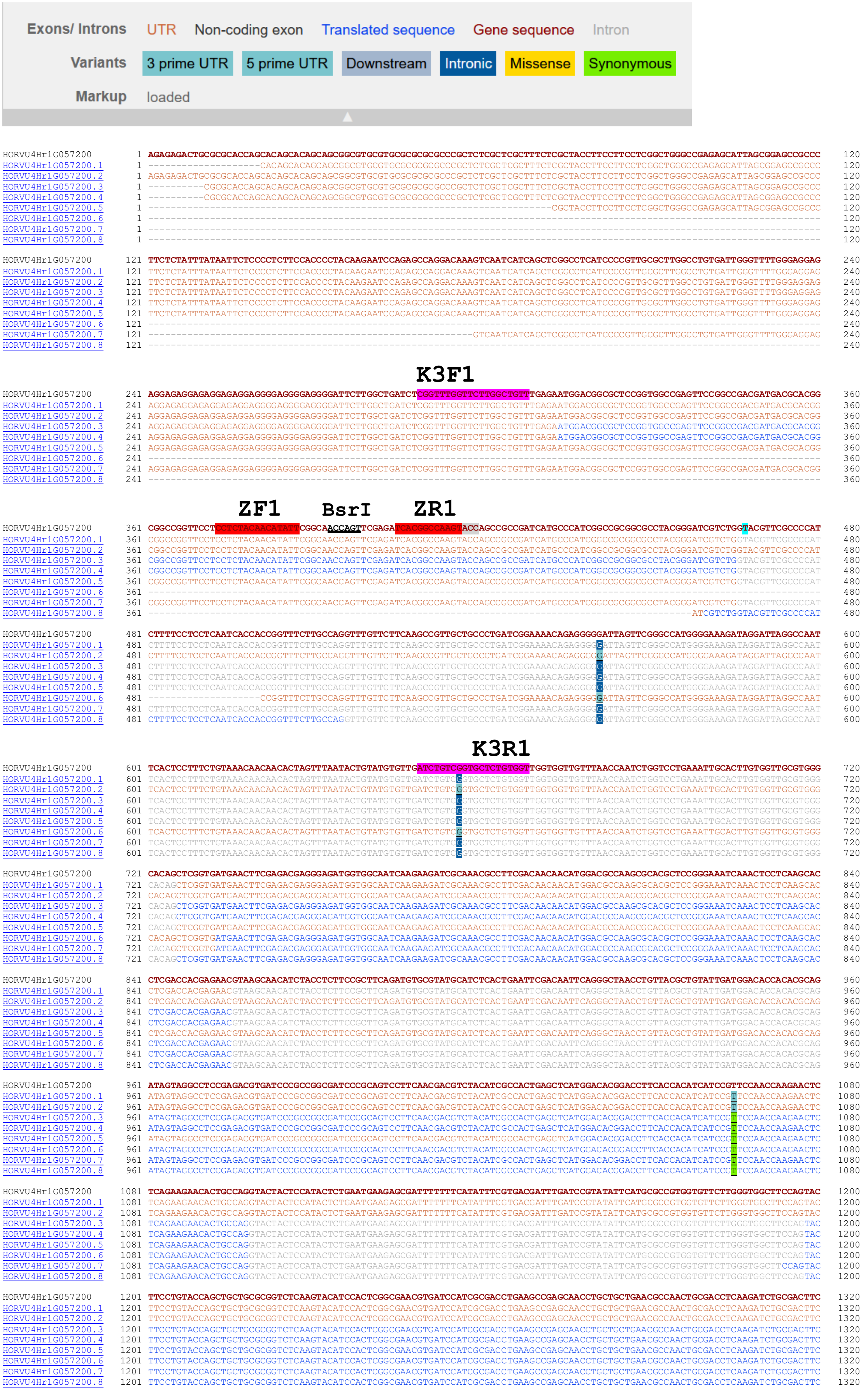
Exons/ Introns
UTR
Non-coding exon
Translated sequence
Gene sequence
Intron
Variants
3 prime UTR
5 prime UTR
Downstream
Intronic
Missense
Synonymous
Markup
loaded
K3F1
ZF1
BsrI
ZR1
K3R1

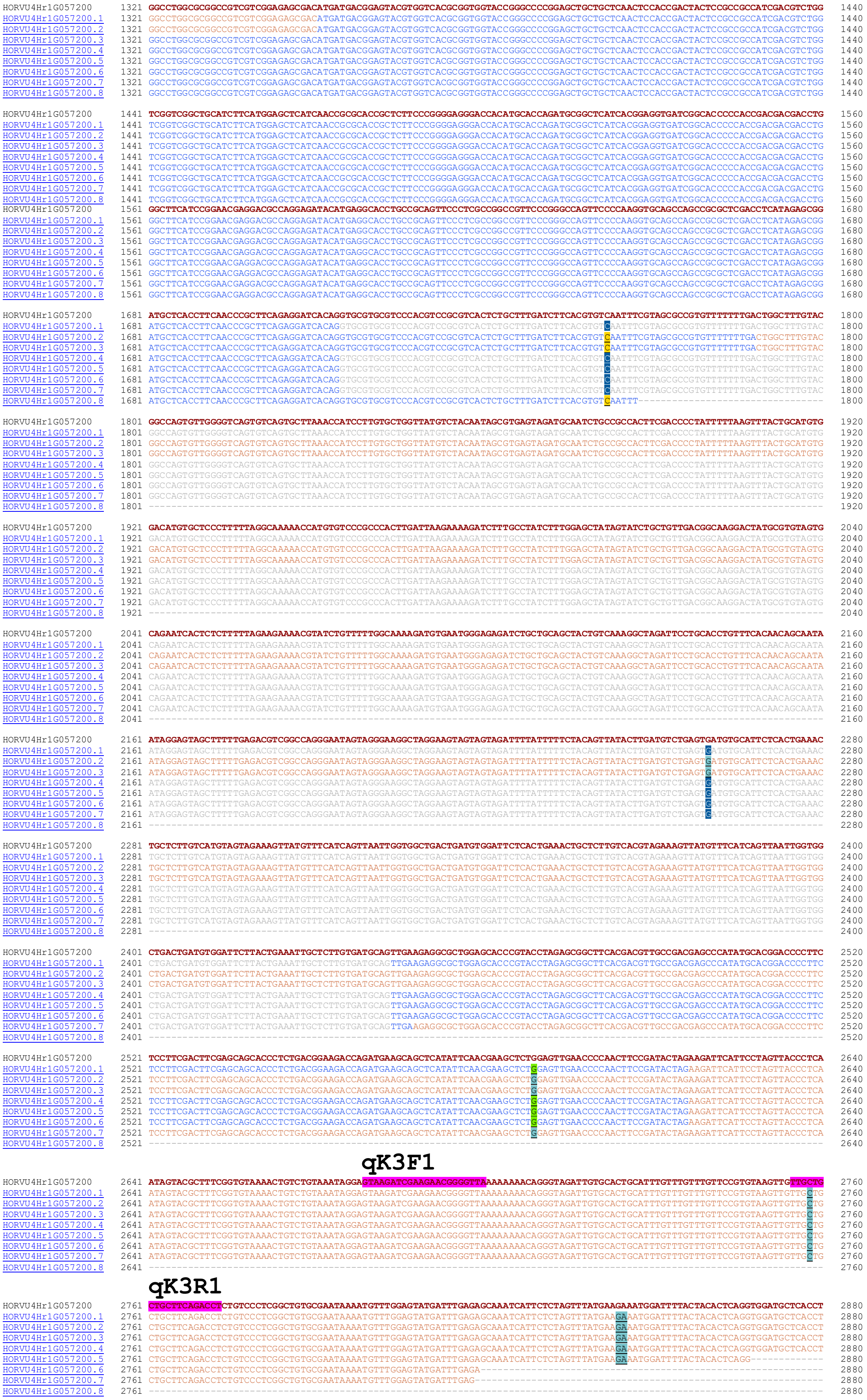
qK3F1
qK3R1

```
HORVU4Hr1G057200     2881 GATATTGAGAATGCAGTTTATGTTTCTATAGATGAAAGGCAGACGTATTTAGGATCACTATGTTTC 2946
HORVU4Hr1G057200.1   2881 GATATTGAGAATGCAGTTTATGTTTCTATAGATGAAAGGCAGACGTATTTAGGATCACTATGTTTC 2946
HORVU4Hr1G057200.2   2881 GATATTGAGAATGCAGTTTATGTTTCTATAGATGAAAGGCAG------------------------ 2946
HORVU4Hr1G057200.3   2881 GATATTGAGAATGCAGTTTATGTTTC---------------------------------------- 2946
HORVU4Hr1G057200.4   2881 GATATTGAGAATGCAGTTTATGTTTC---------------------------------------- 2946
HORVU4Hr1G057200.5   2881 ------------------------------------------------------------------ 2946
HORVU4Hr1G057200.6   2881 ------------------------------------------------------------------ 2946
HORVU4Hr1G057200.7   2881 ------------------------------------------------------------------ 2946
HORVU4Hr1G057200.8   2881 ------------------------------------------------------------------
```

Figure S1. Transcript comparison of the HORVU4Hr1G057200 *HvMPK3* gene**.** Aligned splicing variants of the *HvMPK3* gene were downloaded from the transcript comparison view of the EnsemblPlants *HvMPK3* gene model HORVU4Hr1G057200. Colour code of the individual features of the gene model is shown above the alignment. Z1 TALEN pair binding sites are indicated with red boxes. Annealing positions of the K3F1/K3R1 and qK3F1/qK3R1 primer pairs are shown in purple boxes. K3F1/K3R1 primers were used for the PCR amplification of the 380 bp genomic DNA fragment covering Z1 TALEN binding sites, whereas qK3F1/qK3R1 primers were used for the RT-qPCR based quantification of the *HvMPK3* gene expression. Mutations induced by Z1 TALEN pair were genotyped by restriction digestion of the 380 bp PCR amplicons with BsrI and SacII, respectively (PCR-RE). Diagnostic restriction sites of the BsrI endonuclease is underlined.

```
Score             Identities            Positives             Gaps
585 bits(1509)    268/365(73%)          319/365(87%)          0/365(0%)

HvMPK3   3    GAPVAEFRPTMTHGGRFLLYNIFGNQFEITAKYQPPIMPIGRGAYGIVCSVMNFETREMV  62
              G    +F    THGG+F+ Y+IFG+ FEIT+KY+PPI+PIGRGAYGIVCSV++ ET E+V
AtMPK3   5    GGQYTDFPAVETHGGQFISYDIFGSLFEITSKYRPPIIPIGRGAYGIVCSVLDTETNELV  64

HvMPK3   63   AIKKIANAFDNNMDAKRTLREIKLLKHLDHENIVGLRDVIPPAIPQSFNDVYIATELMDT  122
              A+KKIANAFDN+MDAKRTLREIKLL+HLDHENI+ +RDV+PP + + F+DVYI+TELMDT
AtMPK3   65   AMKKIANAFDNHMDAKRTLREIKLLRHLDHENIIAIRDVVPPPLRRQFSDVYISTELMDT  124

HvMPK3   123  DLHHIIRSNQELSEEHCQYFLYQLLRGLKYIHSANVIHRDLKPSNLLLNANCDLKICDFG  182
              DLH IIRSNQ LSEEHCQYFLYQLLRGLKYIHSAN+IHRDLKPSNLLLNANCDLKICDFG
AtMPK3   125  DLHQIIRSNQSLSEEHCQYFLYQLLRGLKYIHSANIIHRDLKPSNLLLNANCDLKICDFG  184

HvMPK3   183  LARPSSESDMMTEYVVTRWYRAPELLLNSTDYSAAIDVWSVGCIFMELINRAPLFPGRDH  242
              LARP+SE+D MTEYVVTRWYRAPELLLNS+DY+AAIDVWSVGCIFMEL+NR PLFPG+DH
AtMPK3   185  LARPTSENDFMTEYVVTRWYRAPELLLNSSDYTAAIDVWSVGCIFMELMNRKPLFPGKDH  244

HvMPK3   243  MHQMRLITEVIGTPTDDDLGFIRNEDARRYMRHLPQFPRRPFPGQFPKVQPAALDLIERM  302
              +HQMRL+TE++GTPT+ DLGF  NEDA+RY+R LP FPR+P    F  V P A+DL++RM
ATMPK6   245  VHQMRLLTELLGTPTESDLGFTHNEDAKRYIRQLPNFPRQPLAKLFSHVNPMAIDLVDRM  304

HvMPK3   303  LTFNPLQRITVEEALEHPYLERLHDVADEPICTDPFSFDFEQHPLTEDQMKQLIFNEALE  362
              LTF+P +RITVE+AL H YL +LHD  DEPIC  PFSF+FEQ PL E+Q+K++I+ EA+
AtMPK6   305  LTFDPNRRITVEQALNHQYLAKLHDPNDEPICQKPFSFEFEQQPLDEEQIKEMIYQEAIA  364

HvMPK3   363  LNPNF  367
              LNP +
AtMPK6   365  LNPTY  369
```

Figure S2. Alignment of the barley HORVU4Hr1G057200.4 HvMPK3 and Arabidopsis AtMPK3 amino acid sequences**.** HORVU4Hr1G057200.4 HvMPK3 (Ensembl Plants) and Arabidopsis AtMPK3 (NCBI code NP_190150.1) were aligned using protein-protein BLAST suite of NCBI (https://blast.ncbi.nlm.nih.gov/Blast.cgi). Parameters of the alignment output are shown above the alignment. HORVU4Hr1G057200.4 HvMPK3 is 369 amino acid long protein and AtMPK3 is 370 amino acid long protein. HvMPK3 amino acids coded by the 26th codon and 27th codon of the *HORVU4Hr1G057200.4 HvMPK3* gene are shown in bolt and are highlighted in yellow. After decoding of these codons frameshifts occur in the Z1 TALEN pair mutated versions (-4 bp, -5bp and -20bp deletions) of the *HORVU4Hr1G057200.4 HvMPK3* gene (see also Figure 1C).

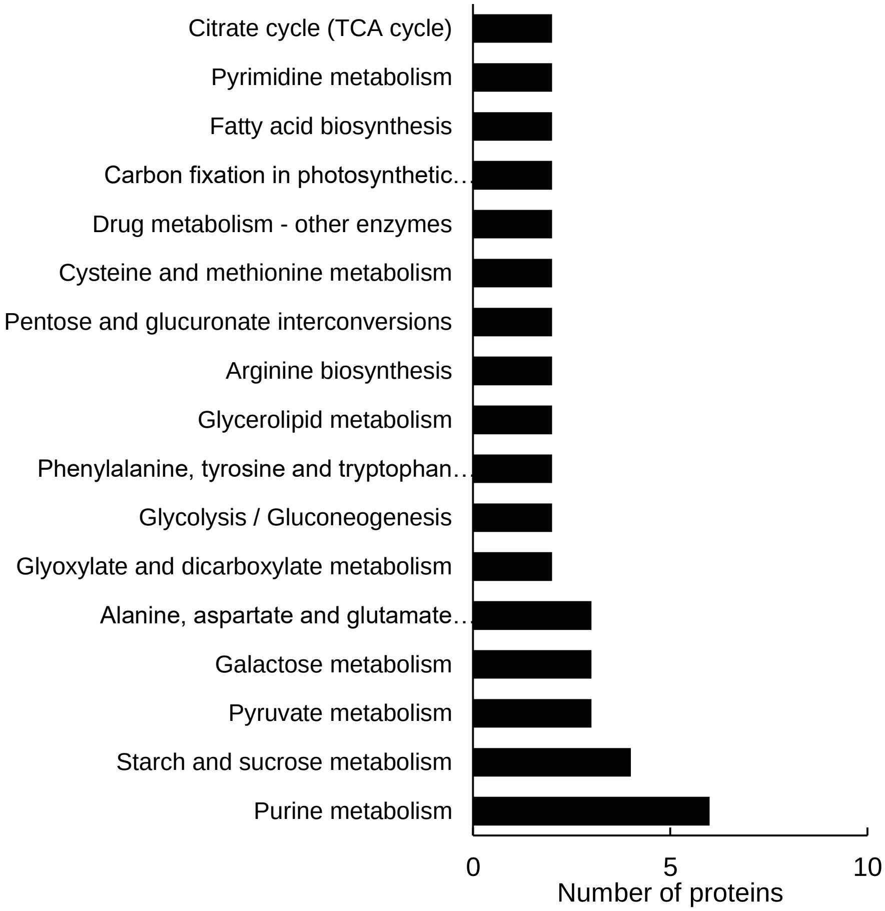

Figure S3. KEGG pathway analysis of differentially regulated proteins found between roots of *HvMPK3* KO lines and WTs.

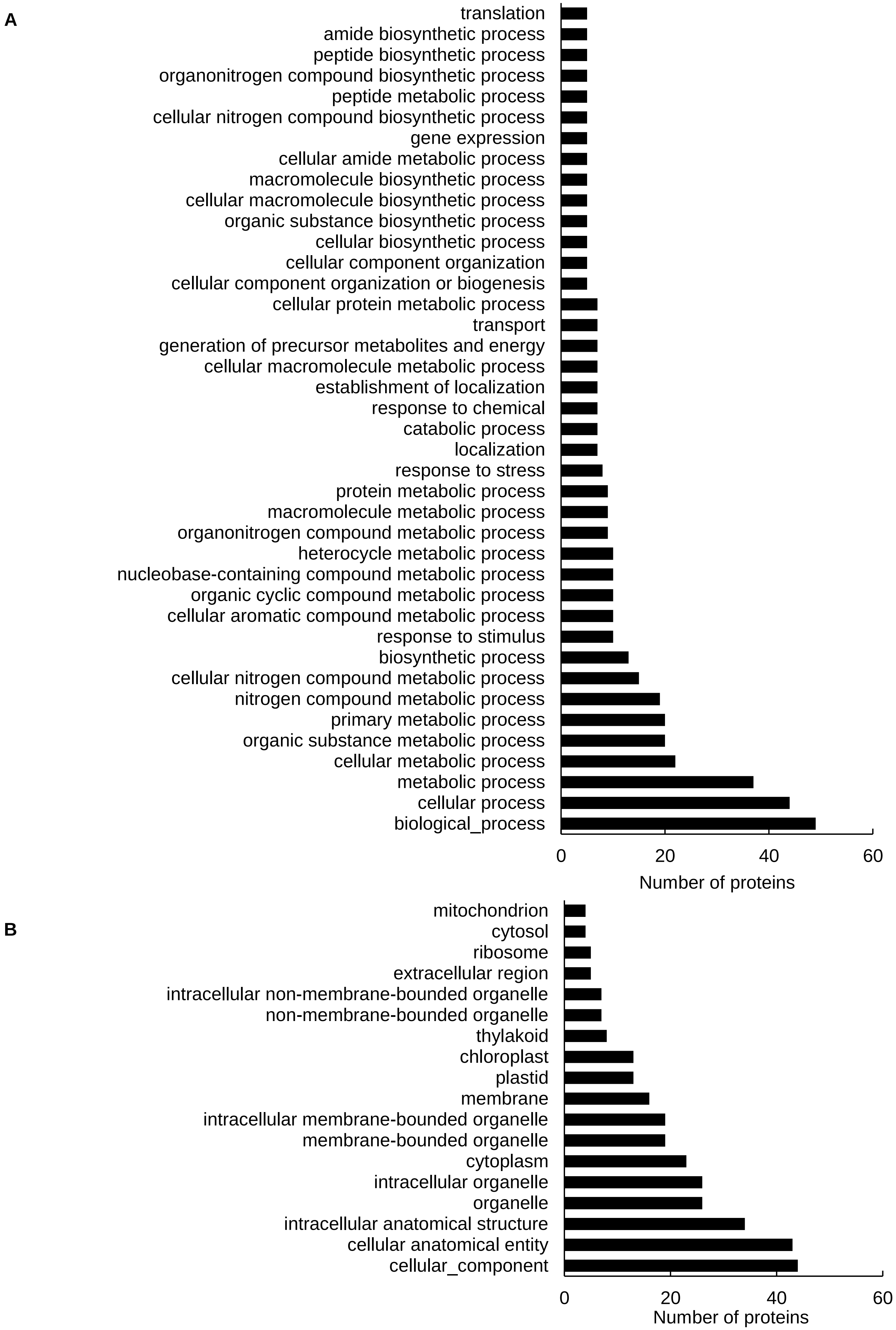

Figure S4. Gene ontology annotation analysis of differentially regulated proteins found between above ground parts of *HvMPK3* KO lines and WTs. (A, B) GO annotation according to biological proces (A) and cell compartment (B)

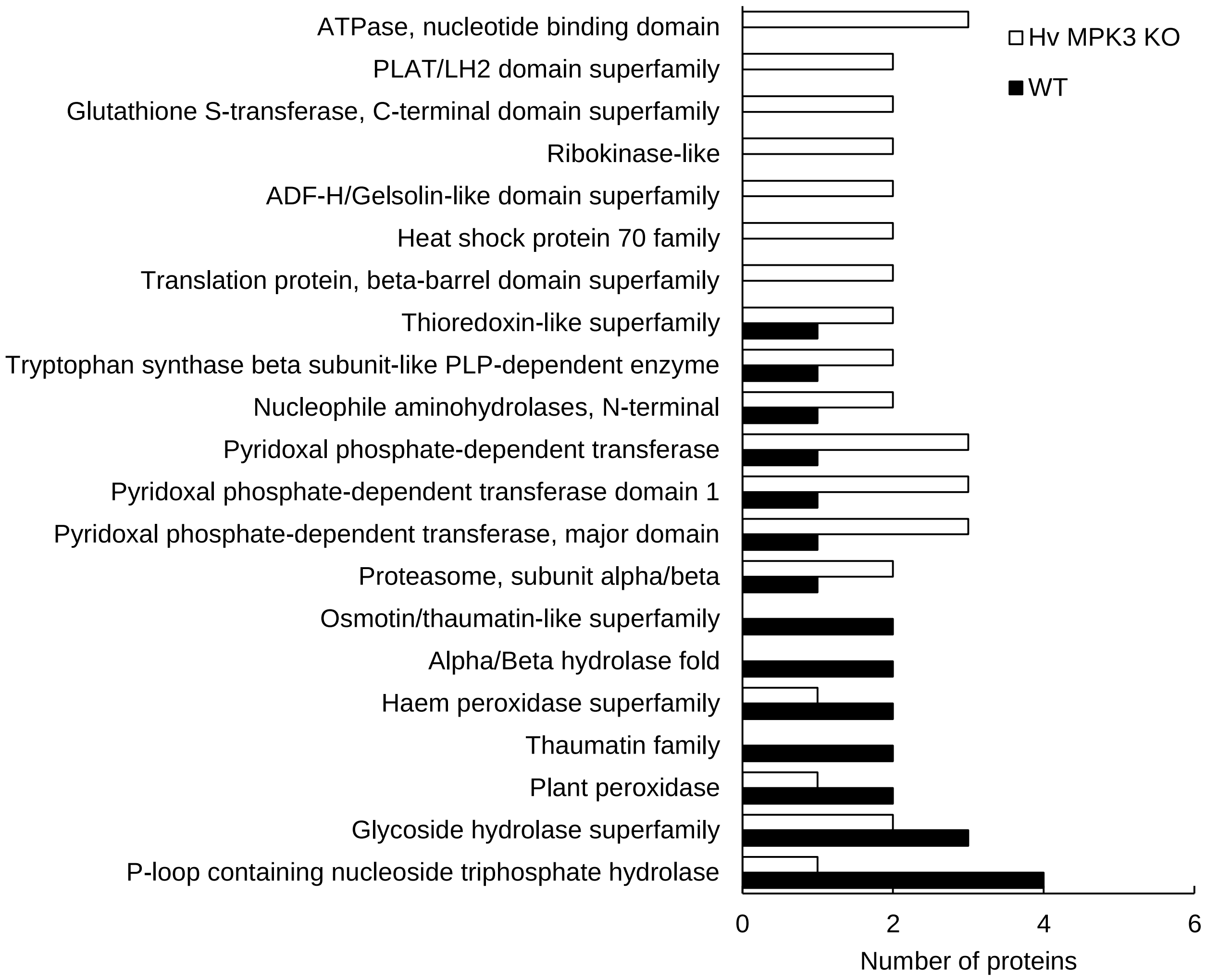

Figure S5. Evaluation of protein families in the differential proteomes of WT and *HvMPK3* KO roots. Graph showing the protein abundances in individual protein families as evaluated by OmicsBox software. Families with differences between WT and *HvMPK3* KO plants are included.

A

B

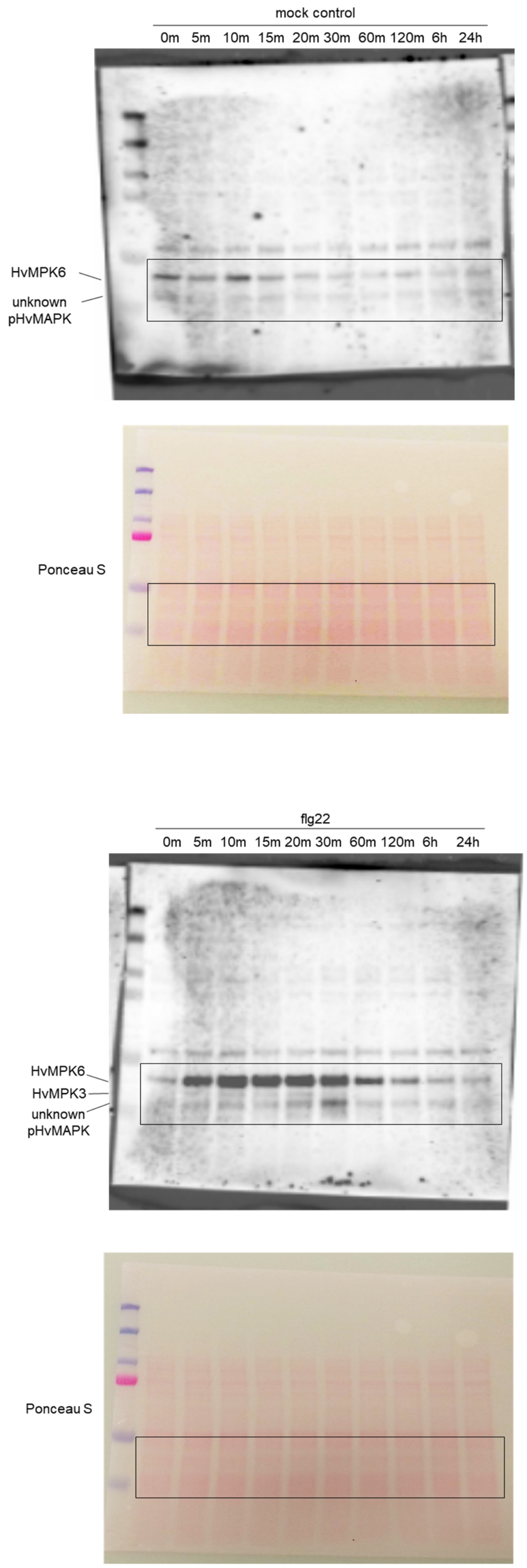

Figure S6. Full scans of the entire original membranes presented in Figure 2A showing activated MAPKs in mock-treated (A) and flg22-treated (B) barley wild type roots.The highlighted region shows the presented section.

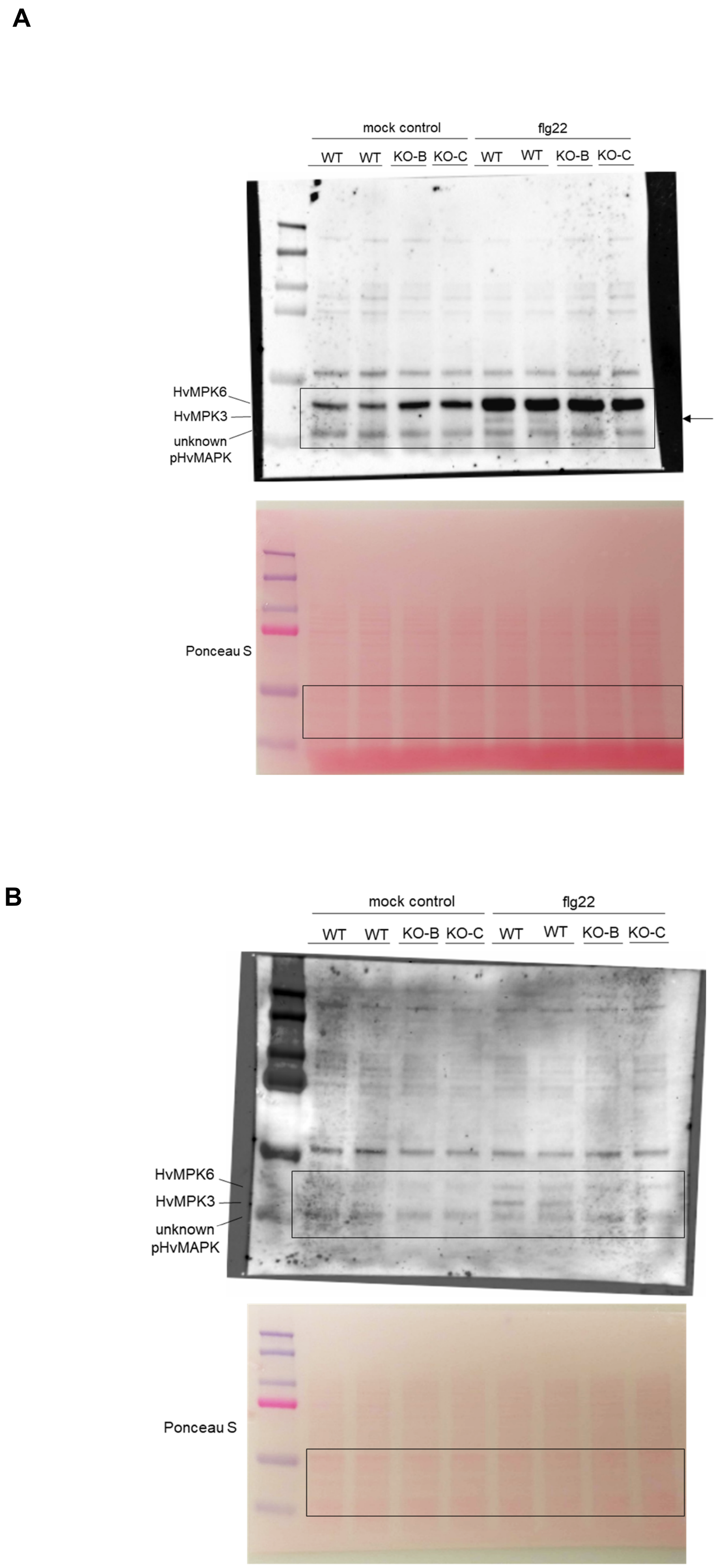

Figure S7. Full scans of the entire original membranes presented in Figure 2C (A) and 2E (B) showing flg22-induced MAPK activation in barley wild types and *HvMPK3* KO roots as found using primary polyclonal (A) and monoclonal (B) anti-pERK antibody. The highlighted region shows the presented section.

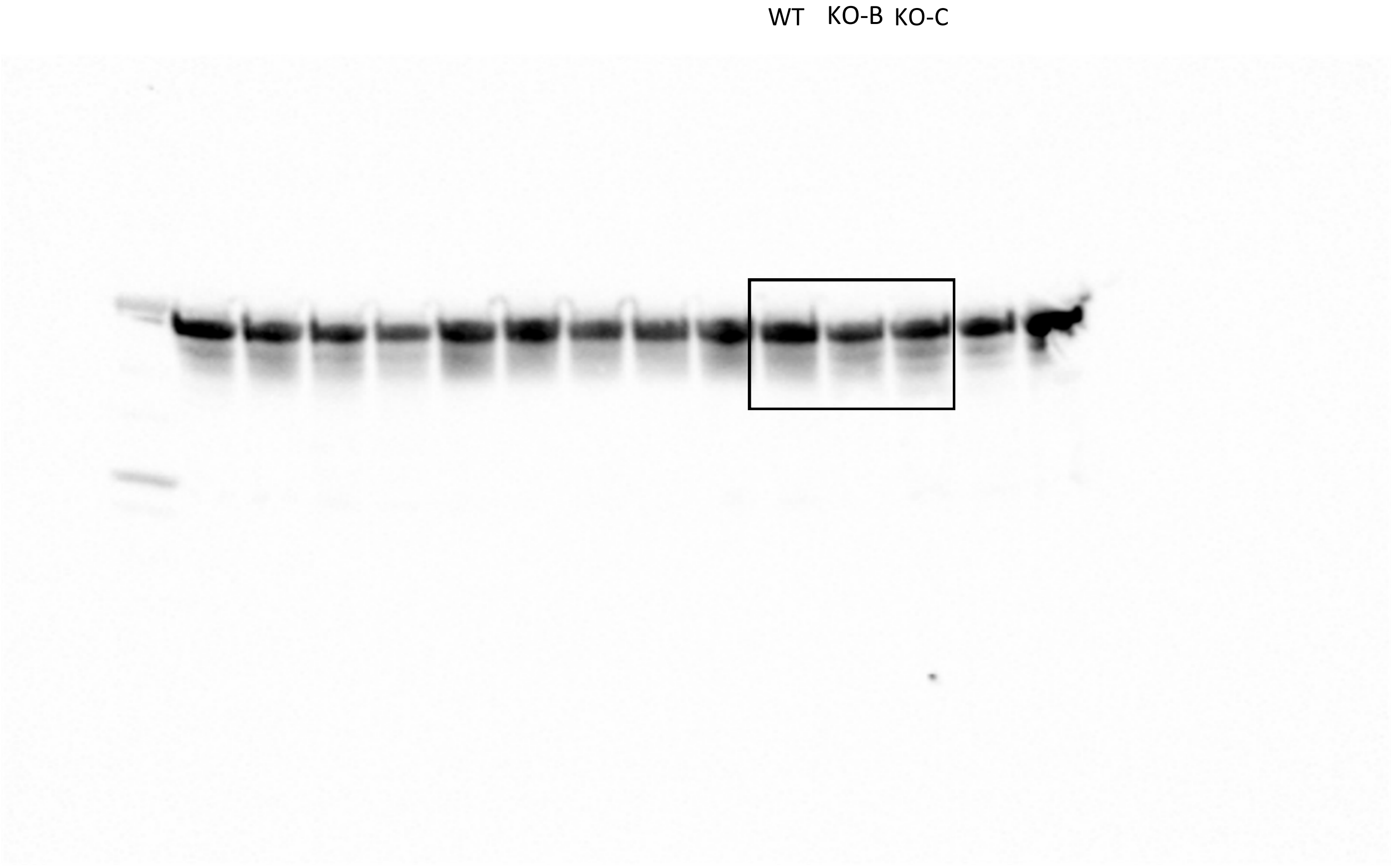

Figure S8. Full scan of the entire original membrane probed with ati-HSP70 primary antibody presented in Figure 4A.The highlighted region shows the presented section. Samples loaded on lanes which are not annotated are not relevant to this this study.

A

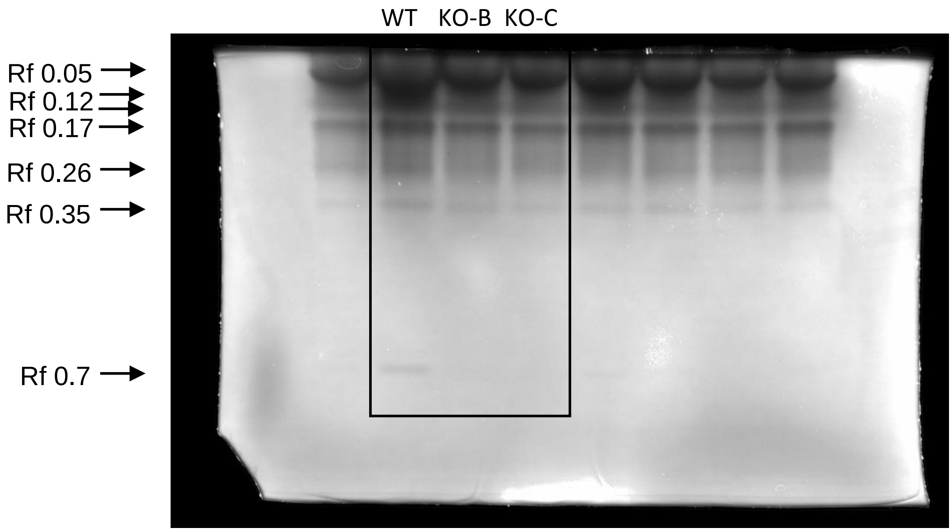

B

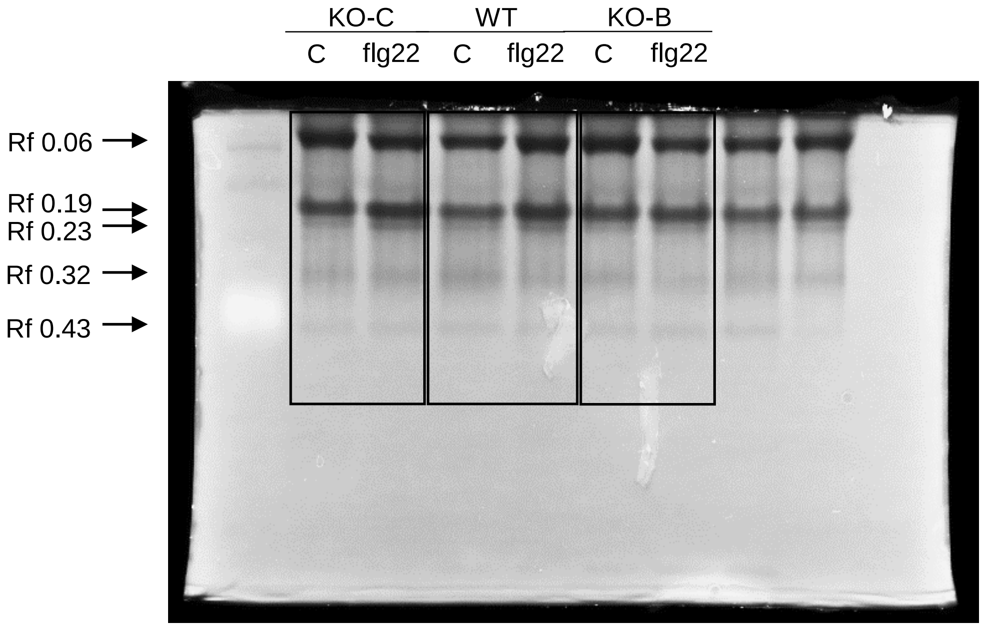

Figure S9. Full scans of the entire original gel stained for activity of chitinases presented in Figures 5A (A) and Figure 8A (B). The highlighted regions shows the presented sections. Samples loaded on lanes which are not annotated are not relevant to this this study.
